# Dual clumped isotope thermometry resolves kinetic biases in carbonate formation temperatures

**DOI:** 10.1038/s41467-020-17501-0

**Published:** 2020-08-10

**Authors:** David Bajnai, Weifu Guo, Christoph Spötl, Tyler B. Coplen, Katharina Methner, Niklas Löffler, Emilija Krsnik, Eberhard Gischler, Maximilian Hansen, Daniela Henkel, Gregory D. Price, Jacek Raddatz, Denis Scholz, Jens Fiebig

**Affiliations:** 1 Institute of Geosciences, Goethe University Frankfurt, Altenhöferallee 1, Frankfurt am Main, 60438 Germany; 2 Department of Geology and Geophysics, Woods Hole Oceanographic Institution, 266 Woods Hole Road, Woods Hole, MA 02543 USA; 3 Institute of Geology, University of Innsbruck, Innrain 52, Innsbruck, 6020 Austria; 4 US Geological Survey, 12201 Sunrise Valley Drive, Reston, VA 20192 USA; 5 Senckenberg Biodiversity and Climate Research Centre (SBiK-F), Senckenberganlage 25, Frankfurt am Main, 60325 Germany; 6 Institute of Geosciences, Johannes Gutenberg University Mainz, Johann-Joachim-Becher-Weg 21, Mainz, 55128 Germany; 7 GEOMAR Helmholtz Centre for Ocean Research, Wischhofstr. 1–3, Kiel, 24148 Germany; 8 School of Geography, Earth and Environmental Sciences, University of Plymouth, Drake Circus, Plymouth, PL4 8AA UK; 9 Present Address: Institute of Geology and Mineralogy, University of Cologne, Zülpicher Str. 49b, Cologne, 50674 Germany

**Keywords:** Biogeochemistry, Palaeoceanography, Palaeoclimate, Physical oceanography

## Abstract

Surface temperature is a fundamental parameter of Earth’s climate. Its evolution through time is commonly reconstructed using the oxygen isotope and the clumped isotope compositions of carbonate archives. However, reaction kinetics involved in the precipitation of carbonates can introduce inaccuracies in the derived temperatures. Here, we show that dual clumped isotope analyses, i.e., simultaneous ∆_47_ and ∆_48_ measurements on the single carbonate phase, can identify the origin and quantify the extent of these kinetic biases. Our results verify theoretical predictions and evidence that the isotopic disequilibrium commonly observed in speleothems and scleractinian coral skeletons is inherited from the dissolved inorganic carbon pool of their parent solutions. Further, we show that dual clumped isotope thermometry can achieve reliable palaeotemperature reconstructions, devoid of kinetic bias. Analysis of a belemnite rostrum implies that it precipitated near isotopic equilibrium and confirms the warmer-than-present temperatures during the Early Cretaceous at southern high latitudes.

## Abstract (web summary)

Some palaeotemperature proxies suffer from inaccuracies related to kinetic fractionations occurring during carbonate mineral growth. Here, the authors show that dual clumped isotope thermometry can identify the origin of these kinetic biases and allows for the reconstruction of accurate environmental temperatures.

## Introduction

Palaeotemperature reconstructions are fundamental for understanding climatic changes in Earth’s past. For a long time, the oxygen isotope composition (*δ*^18^O) of carbonate remains of marine calcifiers and terrestrial carbonates has served as one of the most widely used palaeotemperature proxies^1–3^. However, to reconstruct carbonate growth temperatures using the oxygen isotope thermometer, the oxygen isotope composition of the (palaeo)fluid from which the carbonate mineral crystallised must be known. Uncertainties in estimating the oxygen isotope composition of these (palaeo)fluids, e.g., ancient seawater or meteoric precipitation, make *δ*^18^O-based temperature reconstructions ambiguous. The carbonate clumped isotope (∆_47_) thermometer overcomes this problem by examining the clumping of ^13^C and ^18^O isotopes within a single carbonate phase^4^ and eliminates the need to know the isotopic composition of the water from which carbonates precipitated. Under thermodynamic equilibrium, the clumped isotope composition of a carbonate only depends on its crystallisation temperature.

However, in addition to temperature and fluid *δ*^18^O, kinetic effects occurring during carbonate (bio)mineralisation can influence the isotopic composition of the carbonates. Departures from oxygen isotope and clumped isotope equilibrium due to kinetic effects pose considerable challenges in obtaining accurate palaeotemperature estimates. Such kinetic departures are present in most Earth-surface carbonates^5^, most notably speleothems^2,6–9^, brachiopod shells^10–13^, and coral skeletons^14–20^. If unaccounted for, these kinetic biases could lead to significant over- or underestimation of the carbonate formation temperatures^6,7,12,16,18,21^. Despite numerous efforts, it remains difficult to identify and correct for these kinetic effects, especially in extinct calcifiers without modern analogues^22–24^.

The ∆_48_ value is a measure of the excess abundance of the ^18^O–^12^C–^18^O isotopologue in a given pool of CO_2_ molecules relative to the stochastic distribution^25^. Theoretical studies suggest that simultaneous measurements of ∆_48_ and ∆_47_ in CO_2_ evolved from phosphoric acid digestion of carbonates, referred to here as dual clumped isotope thermometry, can identify kinetic effects involved in carbonate formation^26,27^. Moreover, this method holds the potential to correct for kinetic biases and derive accurate temperature estimates^26,27^. Precise determination of carbonate ∆_48_ values has not been possible until recent advances in mass spectrometry, due to the very low abundance of ^18^O–^12^C–^18^O, the main isotopologue contributing to the ∆_48_ signal in carbonate minerals^25^.

Here we report simultaneous high precision ∆_47_ and ∆_48_ measurements on representative carbonate samples of different origins and demonstrate the potential of dual clumped isotope thermometry in quantitative palaeotemperature reconstruction by comparing these experimental observations with independent theoretical predictions.

## Results

### The temperature dependence of equilibrium ∆_47_ and ∆_48_ values

**Fig. 1:**
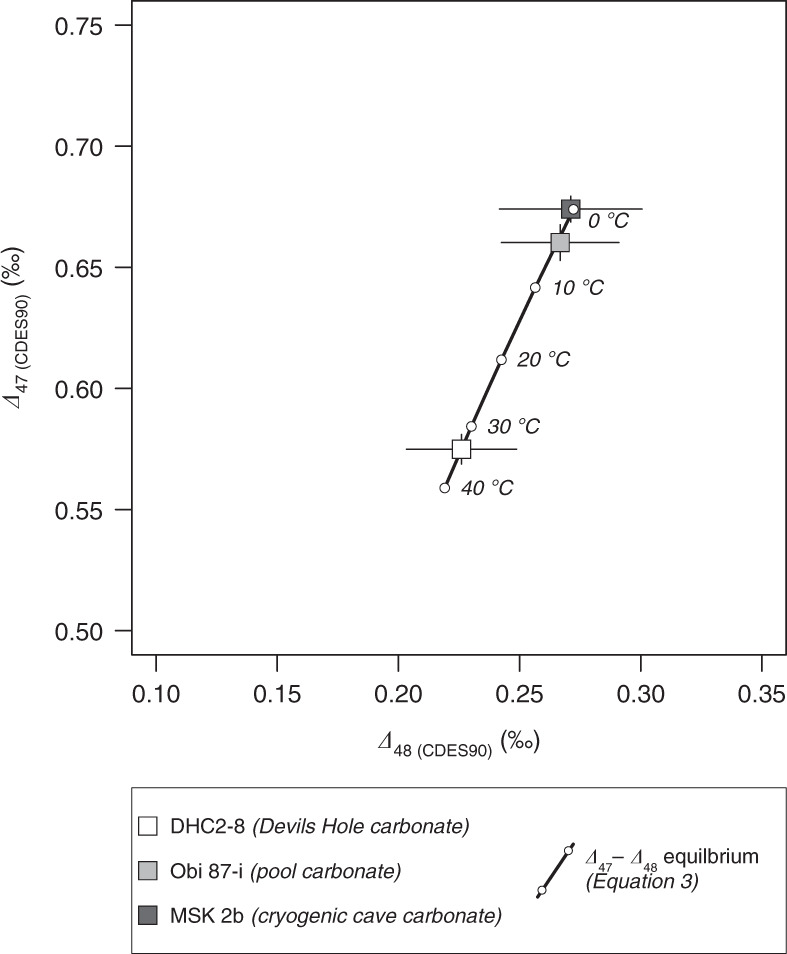
The equilibrium relationship between carbonate ∆_47 (CDES90)_ and ∆_48 (CDES90)_ values. The estimated equilibrium relationship is obtained by combining the empirical ∆_47 (CDES90)_ vs temperature and ∆_48 (CDES90)_ vs temperature relationships after anchoring each to the measured isotopic composition of the Devils Hole carbonate (Eqs. (1–3); see text for details). The fact that the mean ∆_47 (CDES90)_ and ∆_48 (CDES90)_ values of the pool carbonate and the cryogenic carbonate samples, both of which are thought to form very close to isotopic equilibrium (see “Methods” and Supplementary Fig. 1), fall within ±0.001‰ of the expected equilibrium values confirm the robustness of our equilibrium line.

Before examining the potential kinetic isotope effects in our carbonate samples, we constrained the equilibrium ∆_47_ vs ∆_48_ relationship for carbonates by integrating theoretical estimations with experimental measurements (Fig. 1). First, we derived empirical equilibrium ∆_47 (CDES90)_ vs temperature and ∆_48 (CDES90)_ vs temperature relationships by combining the theoretically estimated equilibrium temperature dependence of carbonate ∆_63_ and ∆_64_ values^28^ with our experimentally determined acid fractionation factors (0.194‰ and 0.138‰, respectively, see “Methods”). We chose to use the theoretical calcite calibration for both calcite and aragonite minerals, as no systemic difference in equilibrium ∆_47_ values between the two has been observed in the majority of studies^29^. Secondly, we analysed the clumped isotope composition of a vein calcite from Devils Hole (DHC2-8) and used it to anchor our empirically derived equilibrium ∆_47 (CDES90)_ vs temperature and ∆_48 (CDES90)_ vs temperature relationships. The Devils Hole carbonate is believed to have precipitated extremely slowly (0.1–0.8 μm year^−1^) under stable environmental conditions at 33.7(±0.8) °C, and thus its isotopic composition has been postulated to represent thermodynamic equilbrium^5,30–32^. Specifically, we calculated the differences between the ∆_47 (CDES90)_ and ∆_48 (CDES90)_ values of DHC2-8 and the corresponding values predicted by our empirical relationships at 33.7 °C. We then added these differences (0.010‰ for ∆_47 (CDES90)_ and −0.021‰ for ∆_48 (CDES90)_) to the empirical relationships, leading to the final equilibrium relationships (0–40 °C):

1 $${\it{\Delta }}_{47\,\left( {{\mathrm{CDES90}}} \right)} 	= \, 0.3642 - 2.914\;\times\;10^2/T + 1.800 \, \times\;10^5/T^2 \\ 	\,\,\,\,\,\,- 2.657\;\times\;10^7/T^3 + 1.493\;\times\;10^9/T^4,$$

2 $${\it{\Delta }}_{48\,\left( {{\mathrm{CDES90}}} \right)} 	= \, 0.1742 - 5.897\;\times\;10/T + 1.252\;\times\;10^4/T^2 \\ 	 \,\,\,\,\,\,+\;5.039 \, \times\,10^6/T^3 - 5.631\;\times\;10^8/T^4,$$

where *T* is in K. Lastly, the combination of Eqs. (1) and (2) leads to our estimated equilibrium ∆_47 (CDES90)_ vs ∆_48 (CDES90)_ relationship for carbonates (Fig. 1):

3 $${\it{\Delta }}_{47\,\left( {{\mathrm{CDES90}}} \right)} 	= \, - 0.4771 + 9.102\;\times\;{\it{\Delta }}_{48\,({\mathrm{CDES90}})} - 31.709\;\times\;{\it{\Delta }}_{48\,({\mathrm{CDES90}})}^2 \\ 	\,\,\,\,\,\,+ \;65.561\;\times\;{\it{\Delta }}_{48\,({\mathrm{CDES90}})}^3 - 54.560\;\times\;{\it{\Delta }}_{48\,({\mathrm{CDES90}})}^4.$$

To check whether Eqs. (1–3) indeed represent thermodynamic equilibrium, especially at temperatures below 33.7 °C, we analysed two additional carbonate samples which are thought to have precipitated slowly from their parent solutions and thus may exhibit equilibrium ∆_47_ and ∆_48_ signatures: a cave pool carbonate (Obi 87-i) and a cryogenic cave carbonate (MSK 2b), formed at 4(±1.5) °C and 0 °C, respectively (see “Methods”). In particular, the *δ*^13^C and *δ*^18^O values of the cryogenic carbonate sample suggest crystallisation over the final stage of the freezing process during which isotopic equilibrium is closely approached^33,34^ (Supplementary Table 1 and Supplementary Fig. 1). The ∆_47 (CDES90)_ and ∆_48 (CDES90)_ values of both the pool carbonate and the cryogenic carbonate fall within ±0.001‰ of the equilibrium values calculated using Eqs. (1) and (2) (Fig. 1), strongly supporting the robustness of our equilibrium ∆_47_ and ∆_48_ relationships. Furthermore, our estimated temperature dependence of carbonate ∆_47 (CDES90)_ (Eq. (1)) is indistinguishable (within ±0.010‰) from the ∆_47_ vs temperature relationship derived from laboratory-synthesised carbonates (Supplementary Fig. 2)^29^.

### Carbonates formed in kinetic environments

The clumped isotope composition and the corresponding uncertainties of the carbonate standards and samples analysed in this study are listed in Tables 1 and 2, respectively. In addition to the carbonates that have formed in or close to thermodynamic equilibrium (i.e. the Devils Hole carbonate, the cave pool carbonate, and the cave cryogenic carbonate; see above), we analysed representative carbonates from a variety of kinetic environments (see “Methods”), including a synthetic speleothem, a natural stalagmite (Spannagel Cave, Austria), a warm-water coral (*Porites lutea*), a cold-water coral (*Desmophyllum pertusum*), a modern brachiopod (*Magellania venosa*), and a fossil belemnite (*Belemnopsis* sp.).

**Table 1:** Long-term ∆_47 (CDES90)_ and ∆_48 (CDES90)_ values of carbonate reference materials.

Standard	Replicates	∆_47 (CDES90)_ (‰)	2 SE (1 SD)	∆_48 (CDES90)_ (‰)	2 SE (1 SD)
ETH 1	78	0.212	0.002 (0.010)	0.142	0.008 (0.036)
ETH 2	71	0.212	0.003 (0.011)	0.138	0.007 (0.029)
ETH 3	74	0.615	0.002 (0.010)	0.299	0.010 (0.042)

This table considers data measured during the April–August 2019 measurement period combined with those reported in Fiebig et al.^25^. All replicate analyses from the April–August 2019 measurement period are presented in Supplementary Data 1. The average ∆_47 (CDES90)_ and ∆_48 (CDES90)_ values for the total number (149) of ETH 1 and ETH 2 replicates are 0.212(±0.002)‰ and 0.140(±0.005)‰, respectively. *SE*, standard error; *SD*, standard deviation.

**Table 2:** Clumped isotope composition (∆_47 (CDES90)_ and ∆_48 (CDES90)_) of the samples.

Sample (type)	Replicates	∆_47 (CDES90)_ (‰)	2 SE	∆_48 (CDES90)_ (‰)	2 SE
DHC2-8 (vein calcite)	5	0.575	0.006	0.226	0.023
Obi 87-i (pool carbonate)	7	0.660	0.007	0.267	0.024
MSK 2b (cryogenic cave carbonate)	8	0.674	0.005	0.271	0.029
MHD1 (synthetic speleothem)	6	0.547	0.008	0.254	0.027
SPA121-02 (stalagmite)	6	0.644	0.009	0.290	0.025
PC1_2005 (warm-water coral)	10	0.626	0.005	0.136	0.022
JR (cold-water coral)	10	0.719	0.005	0.189	0.030
Mv143-b (brachiopod)	9	0.675	0.004	0.250	0.015
66-4.65 (belemnite)	9	0.610	0.005	0.251	0.021

This table considers data measured during the April–August 2019, September–December 2019, and January–March 2020 measurement periods. All replicate analyses are presented in Supplementary Data 2–4. The *δ*^18^O and *δ*^13^C values of the samples are reported in Supplementary Table 1.

**Fig. 2:**
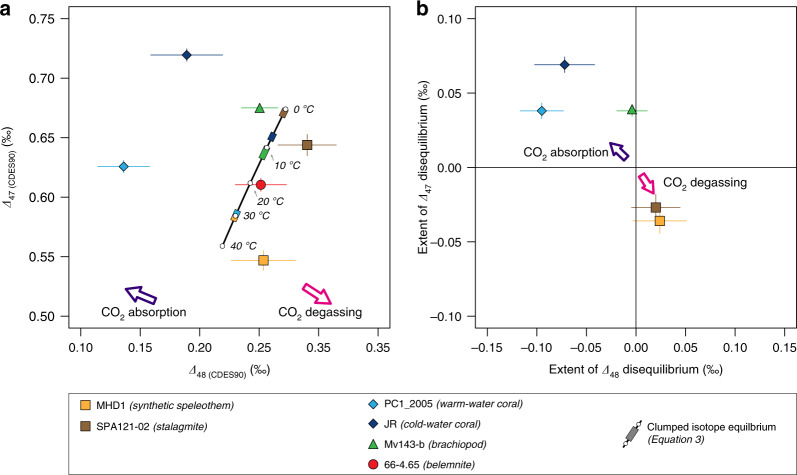
The nature and extent of kinetic isotope effects in representative carbonates. **a** The isotopic compositions of the stalagmite, synthetic speleothem, cold-water coral, warm-water coral, and the brachiopod deviate significantly from their respective equilibrium compositions (coloured rectangles on the equilibrium line), while the belemnite is indistinguishable from the equilibrium line. **b** The cold- and warm-water corals show positive ∆_47 (CDES90)_ offsets and negative ∆_48 (CDES90)_ offsets from the thermodynamic equilibrium, which are consistent with kinetic effects derived from CO_2_ absorption (purple arrow). In comparison, the stalagmite and the synthetic speleothem show negative ∆_47 (CDES90)_ offsets and positive ∆_48 (CDES90)_ offsets that are consistent with kinetic effects derived from CO_2_ degassing (pink arrow). Disequilibrium offsets were calculated relative to the equilibrium ∆_47 (CDES90)_ and ∆_48 (CDES90)_ values expected at the corresponding carbonate growth temperatures (Eqs. (1–3)). All error bars depict 2 standard errors (95% confidence interval^35^).

The mean clumped isotope values of our analysed samples range from 0.547‰ to 0.719‰ for ∆_47 (CDES90)_ values, and from 0.136‰ to 0.290‰ for ∆_48 (CDES90)_ values (Fig. 2a, Table 2, Supplementary Fig. 2). Except for the belemnite sample, which plots within 2 SE (95% confidence interval^35^) of the equilibrium ∆_47 (CDES90)_ vs ∆_48 (CDES90)_ line (Eq. (3)), all the other investigated carbonate samples show significant offsets from the expected clumped isotope equilibrium (Fig. 2, Supplementary Fig. 2). Both the synthetic speleothem and the stalagmite plot below the ∆_47 (CDES90)_ vs ∆_48 (CDES90)_ equilibrium line, with offsets of −0.036‰ and −0.030‰ in the ∆_47 (CDES90)_ values and +0.024‰ and +0.018‰ in the ∆_48 (CDES90)_ values, respectively. In contrast, the two modern corals and the brachiopod plot above the equilibrium line, with offsets ranging from +0.038‰ to +0.069‰ in ∆_47 (CDES90)_ and from −0.004‰ to −0.095‰ in ∆_48 (CDES90)_.

## Discussion

If clumped isotope equilibrium was attained in all samples, their ∆_47 (CDES90)_ and ∆_48 (CDES90)_ values should correlate, due to the exclusive temperature dependence of clumped isotope equilibrium. The fact that the ∆_47 (CDES90)_ and ∆_48 (CDES90)_ values of our investigated samples do not follow such a simple trend provides the most substantial evidence so far that most of these carbonates did not form under isotopic equilibrium (Fig. 2a, Supplementary Fig. 2). Kinetic departures from isotopic equilibrium in the carbonate–water system generally arise from two processes: the slow conversion between dissolved carbon dioxide (CO_2 (aq)_) and bicarbonate (HCO_3_^−^) through (de)hydration/(de)hydroxylation reactions (Eqs. (4) and (5), respectively)^36^ and the incorporation of dissolved inorganic carbon species (DIC) into the carbonate crystal lattice^37,38^.

4 $${\mathrm{CO}}_{{\mathrm{2(aq)}}} + {\mathrm{H}}_{\mathrm{2}}{\mathrm{O}} \leftrightarrow {\mathrm{H}}^ + + {\mathrm{HCO}}_{\mathrm{3}}^ -$$

5 $${\mathrm{CO}}_{{\mathrm{2(aq)}}} + {\mathrm{OH}}^ - \leftrightarrow {\mathrm{HCO}}_{\mathrm{3}}^ -$$

While DIC incorporation seems to introduce only negligible departures from clumped isotope equilibrium^32,39,40^, kinetics associated with the (de)hydration/(de)hydroxylation reactions are predicted to introduce large disequilibrium signatures in the ∆_63_ and ∆_64_ values of the DIC^26,27^. These disequilibrium ∆_63_ and ∆_64_ values of the DIC are expected to be directly transcribed to the ∆_47_ and ∆_48_ values of the carbonates when carbonates precipitate quickly from the solution.

**Fig. 3:**
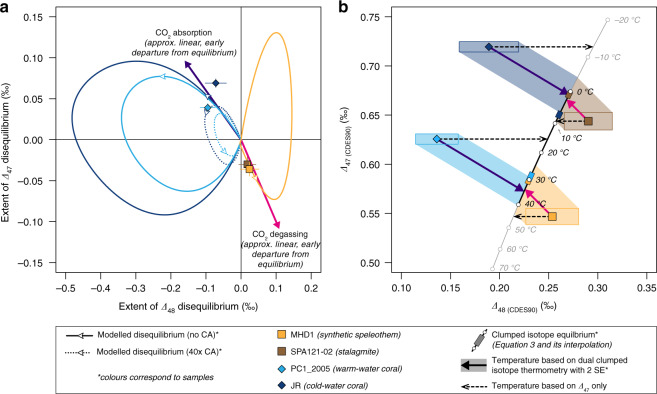
Agreements between model predictions and measured data allow accurate reconstruction of carbonate formation temperatures despite kinetic bias. **a** The deviations from clumped isotope (∆_47_ and ∆_48_) equilibrium observed in the warm- and cold-water corals and the synthetic speleothem agree with the model-predicted kinetic departures^26,27^. Carbonic anhydrase accelerates oxygen isotope and clumped isotope exchange among different DIC species during CO_2_ absorption. The white arrows on the model lines depict the temporal evolution of the predicted disequilibrium effects and are positioned to mark *t* = 500 s in the CO_2_ absorption model simulations and *t* = 170 s (i.e., when 50% of carbonate precipitation proceeded) in the speleothem model simulation, respectively (Methods, Supplementary Data 5). Note, that unlike the speleothem model that simulates the exact formation of our synthetic speleothem sample, the CO_2_ absorption model is a simplification of coral calcification process and thus the timing information in the model has no direct implication on the timescale of coral calcification process. The models predict an almost linear correlation between ∆_47_ and ∆_48_ disequilibrium during the early stage of CO_2_ absorption and CO_2_ degassing, with slopes of −0.6 and −1.0, respectively (the purple and the pink arrows; Supplementary Data 5). **b** Kinetic effects lead to significant underestimation of the coral growth temperature but overestimation of the speleothem formation temperatures based on their ∆_47_ compositions alone (horizontal, dashed arrows). However, using the model-predicted kinetic slopes, i.e., −0.6 for corals (purple arrows) and −1.0 for speleothems (pink arrows), the isotopic composition of these samples can be projected onto the ∆_47_ vs ∆_48_ equilibrium curve to derive more accurate estimates of carbonate formation temperatures^26,27^. Temperatures estimated this way are devoid of kinetic bias and agree within 2 SE with the actual carbonate growth temperatures. All error bars and rectangles depict 2 standard errors (95% confidence interval^35^).

Specifically, under open-system conditions, the evolution of the clumped isotope composition of the DIC follows nonlinear trajectories in ∆_47_ vs ∆_48_ space^26^. For example, for an aqueous solution starting from isotopic equilibrium, the carbonate clumped isotope compositions will increasingly deviate from equilibrium during CO_2_ degassing and CO_2_ absorption, i.e., when dehydration/dehydroxylation reactions dominate over the hydration/hydroxylation reactions, and vice versa (Eqs. 4 and 5). However, as the forward reaction progresses and the product concentration increases, the backward reaction will become more and more significant until the forward and backward reaction rates become identical and the isotopic equilibrium is attained again (Fig. 3a)^26,27^. The exact pattern and extent of these disequilibrium effects during CO_2_ absorption and CO_2_ degassing depend on several parameters^26,27^, such as solution temperature, pH, the isotopic compositions of the gaseous CO_2_ and the solution, and for calcifying organisms, carbonic anhydrase activity that facilitates the interconversion between dissolved carbon dioxide and bicarbonate (Fig. 3a).

Most carbonates in our study exhibit systematic departures from the clumped isotope equilibrium line (Eqs. (1–3)) that are consistent with the model predictions^26,27^, supporting that CO_2_ absorption and CO_2_ degassing reactions are the primary drivers for the observed departures from isotopic equilibrium (Fig. 3a). During scleractinian coral biomineralisation, carbon dioxide derived from metabolic respiration is transformed to bicarbonate via hydration/hydroxylation reactions (Eqs. (4) and (5)) in the calcifying fluid and then subsequently to carbonate ion (CO_3_^2−^) from which the solid carbonate skeleton precipitates^14,26^. In the early stage of CO_2_ absorption, the ∆_47_ values of coral skeletons are predicted to show a positive departure from the expected equilibrium values while their ∆_48_ values show a negative offset (Fig. 3a, Supplementary Data 5). Such a pattern is observed in both the warm-water and cold-water corals in our study (Fig. 2). Moreover, the observed offsets from the respective equilibrium ∆_47_ and ∆_48_ values co-vary and plot close to the model predictions where the CO_2_ absorption kinetics dominate (Fig. 3a, see “Methods”). Similar to the two scleractinian corals, the isotopic composition of the fast-growing brachiopod (*Magellania venosa*) plots above the ∆_47_ vs ∆_48_ equilibrium line (Fig. 2a), showing a positive offset in ∆_47_ but only a minimal negative offset in ∆_48_ (Fig. 2b). The direction of such departures from equilibrium is consistent with the disequilibrium initiated by hydration/hydroxylation reactions^26^, supporting earlier suggestions that hydration and hydroxylation reactions are the dominant causes of the observed clumped isotope disequilibrium in modern brachiopods^12^.

Speleothem precipitation, unlike biomineralisation, is induced by the degassing of CO_2_ from aqueous solutions. The removal of CO_2_ from the very thin solution film on the surface of a speleothem, i.e., the transformation of bicarbonate to dissolved carbon dioxide via dehydration/dehydroxylation reactions (backward reactions in Eqs. (4) and (5)), increases the saturation state of calcium carbonate and leads to carbonate precipitation^6,27,41,42^. In the early stages of CO_2_ degassing, carbonate ∆_47_ values are expected to show a negative offset and ∆_48_ values to show a positive offset from equilibrium^27^ (Fig. 3a, Supplementary Data 5). This pattern is observed in the isotopic compositions of both the synthetic speleothem and the stalagmite samples, which plot below the clumped isotope equilibrium line (Fig. 2). Moreover, the ∆_47_ and ∆_48_ offsets observed in the synthetic speleothem quantitatively agree with the model prediction that explicitly simulates this carbonate formation^27^ (Fig. 3a, see “Methods”).

The agreements between the measured carbonate clumped isotope compositions and our model simulations suggest that dual clumped isotope analysis can be used to identify the dominant reactions involved in carbonate (bio)mineralisation. Furthermore, when combined with numerical modelling, coupled ∆_47_ and ∆_48_ analysis shall allow a more accurate reconstruction of the carbonate formation environment such as temperature, pH, and carbonic anhydrase activity^26,27^. In particular, disequilibrium offsets observed in the two scleractinian corals and the natural and synthetic speleothems fall close to the initial linear segment of the model-predicted departure from isotopic equilibrium, whose slope is relatively insensitive to temperature, pH, and carbonic anhydrase activity^26^ (Fig. 3a). This opens up a unique opportunity to accurately reconstruct carbonate formation temperatures even if the measured ∆_47_ and ∆_48_ values were affected by kinetic effects^26,27^. Under the conditions specific to the formation of our samples (see “Methods”), the model predicts approximately linear correlations between disequilibrium ∆_47_ and ∆_48_ values in carbonates formed during the early stages of CO_2_ degassing and CO_2_ absorption, with slopes of −1.0 and −0.6, respectively (Fig. 3a, Supplementary Data 5). Projecting the measured carbonate isotope composition to the equilibrium ∆_47_ and ∆_48_ line (Eq. (3)) along these predicted linear kinetic trajectories, shall yield their actual formation temperatures, unbiased by kinetic effects^26,27^. Specifically, this leads to temperature estimates of 34(±9) °C, 2(±7) °C, 1(±5) °C, and 34(±6) °C for the synthetic speleothem, the stalagmite, the cold-water coral, and the warm-water coral, respectively (Fig. 3b). These temperature estimates are indistinguishable within 2 SE from the actual formation temperatures of these carbonates, i.e., 30.7(±0.3) °C, 0.0(±2.0) °C, 7.2(±1.0) °C, and 29.3(±1.0) °C, respectively. In contrast, estimates based on the measured ∆_47_ data alone would result in inaccurate and unreasonable temperatures (Fig. 3b). For example, for the synthetic speleothem, the observed disequilibrium ∆_47_ effect of −0.036‰ is similar to that observed in some natural systems^43^, and would yield a temperature that is 10–18 °C too warm compared to its actual formation temperature. Similarly, ∆_47_ thermometry would overestimate the formation temperatures of the stalagmite sample by 4–14 °C, while the ∆_47_-derived temperatures of the warm-water and the cold-water corals would be lower than their actual growth temperatures by 11–17 °C and 18–22 °C, respectively.

For the modern brachiopod specimen, however, even after correction using a model-predicted kinetic slope of −0.6 for CO_2_ absorption, one would still underestimate its growth temperature by 4–13 °C. Compared to scleractinian coral calcification, the mechanism of brachiopod calcification is less explored^12,13^. The current model simulations of CO_2_ absorption were designed based on the mechanism of coral calcification and thus may not have captured the primary sources of isotopic disequilibrium in brachiopods. More studies on specimens of known growth temperatures are required to evaluate the full potential that dual clumped isotope thermometry holds for accurate palaeotemperature reconstructions. However, if it were demonstrated that isotopic compositions of most biogenic carbonates and speleothems follow the early, linear trajectory of departure from equilibrium, accurate palaeotemperature reconstructions would become possible on well-preserved samples even if they are affected by kinetic effects.

The dual clumped isotope analysis also makes it possible to evaluate the extent of isotopic disequilibrium in fossil carbonates, even if the carbonate’s growth temperature and the isotopic composition of its parent water are not known. This, for the first time, offers the opportunity to investigate if the isotopic composition of extinct calcifiers was affected by kinetic effects. The isotopic composition of the Cretaceous belemnite sample falls within 2 SE of the ∆_47_ vs ∆_48_ equilibrium line (Fig. 2a), suggesting that it precipitated indistinguishable from thermodynamic equilibrium at a temperature of 20.5(±1.9) °C. This temperature estimate supports southern high-latitude warmth during the Early Cretaceous^44,45^.

More generally, our finding that the belemnite did but the brachiopod did not form in isotopic equilibrium has significant implications for the palaeotemperature estimates derived from the oxygen isotope compositions of these two archives. Multiple studies have reported colder *δ*^18^O-derived belemnite palaeotemperatures compared to coeval brachiopod-based temperature estimates, when using the same *δ*^18^O vs temperature relationship for both taxa and assuming a constant oxygen isotope composition for the seawater^1,3,46,47^. To reconcile this discrepancy, it was proposed that belemnites migrated to colder waters^47,48^. However, based on the most current assessment of belemnite palaeobiology^49^, both long-distance and vertical migration to significantly colder waters is unlikely for these animals. Belemnites presumably lived in the upper 200 m of the water column and were mostly restricted to continental shelves. Our results thus support that the apparent warm temperature estimates derived from brachiopod *δ*^18^O reflect the kinetic bias in their isotopic compositions^12^.

We have demonstrated that the dual clumped isotope method, i.e., the simultaneous measurement of carbonate ∆_47_ and ∆_48_ values in a single phase, makes it possible to identify carbonates that did not precipitate in thermodynamic equilibrium from their parent water. The comparison of the measured isotopic compositions with theoretical predictions enabled us to pinpoint the dominant kinetic processes responsible for the isotopic disequilibrium and to reconstruct carbonate formation temperatures accurately. Our results highlight the potential that dual clumped isotope thermometry holds for accurate palaeoclimate reconstructions and the identification of the kinetic fractionation processes dominant in carbonate (bio)mineralisation.

## Methods

### Samples

Devils Hole vein calcite: A Holocene vein calcite (DHC2-8) that precipitated 4.5–16.9 ka before present, was collected from the Devils Hole cave #2 in Nevada, USA (36.427138 N, 116.291172 W). It is postulated that DHC2-8 precipitated at extremely slow rates, i.e., 0.1–0.8 μm year^−1^, at a constant temperature of 33.7(±0.8) °C^30^. For this study, we prepared a ca 0.5 × 0.5 × 1.5 cm slab of calcite. First, we abraded the surface ca 0.5 mm of the slab with a slow-speed hand-held drill to remove impurities. Then, the slab was cleaned in de-ionised water in an ultrasonic bath for 5 min and dried in a vacuum oven at 30 °C before it was crushed and homogenised using an agate mortar and pestle. Material from the same vein was analysed for clumped isotopes in other studies^5,32^.

Cryogenic cave carbonate: A coarsely crystalline cryogenic cave calcite (MSK 2b) was obtained from Mitterschneidkar Eishöhle in the Austrian Alps (47.1165 N, 11.7407 E). The cave opens at 2258 m above sea level and contained perennial ice in the near-entrance part until 2007. Today the cave is ice-free, and the mean annual air temperature in the interior of the cave is 0.23 °C^33^. Coarsely crystalline cryogenic cave carbonates generally precipitate slowly and very close to 0 °C, otherwise powder-like fine-crystalline cryogenic cave carbonates form^50^. Cryogenic cave carbonates occur in several places in the inner part of the cave, and U–Th dating of these carbonates suggests the presence of perennial ice up to about 2600 years before present^33^. The sample crystals were crushed and homogenised using an agate mortar and pestle and were subsequently dried in a vacuum oven at 30 °C before isotope analysis. Additional information on the potential equilibrium nature of this sample is found in Supplementary Fig. 1.

Cave pool carbonate: A 3.5-cm thick subaqueous calcite sample (Obi 87-i) was collected in 2008 from a perennial pool (Silbersee) in Rasslsystem cave, which is part of the Obir Caves (46.5102 N, 14.5480 E), a series of karst caves in the Southern Alps of Austria, located at approximately 1100 m above sea level. The Obir Caves are hypogene in origin^51^, i.e., they were not connected to the surface and hence had a very stable microclimate until discovered during mining activities in the 1870s. The Silbersee pool, located in the inner part of Rasslsystem cave, has a surface area of 7 × 4 m and is on average ca 1 m deep. The pool water temperature between 1998 and 2002 was 5.4(±0.1) °C, closely corresponding to the long-term mean annual air temperature outside the cave at this elevation^52^. The sample analysed in this study is a 4 mm wide subsample retrieved from 2.7 cm above the base of Obi 87, and is estimated to have formed at about 1500 years before present, based on the U–Th dating of a lower layer in Obi 87 (a layer estimated to have formed about 3800 years before present at 1.5 cm above the base of Obi 87) and assuming a constant calcite growth rate of 5.3 μm year^−1^ (unpublished data, C Spötl). Although the water temperature about 1500 years before present is not precisely known, it likely corresponded to the mean annual air temperature outside the cave at that time in a similar way as the modern pool temperature does. Various temperature proxy data for the Alps suggest that the mean annual air temperature fluctuated by up to ±1.5 °C in the last two millennia before the industrial revolution^53^. Considering the ca 1.5 °C warming in the Alps during the past century, we estimate the water temperature of Silbersee pool from which Obi 87-i precipitated ca 1500 years ago to be 4.0(±1.5) °C. Experiments demonstrated that subaqueous pool carbonates can precipitate in oxygen isotope equilibrium with water^54^. Prior to isotope analyses, Obi 87-i was cleaned in de-ionised water in an ultrasonic bath, crushed and homogenised using an agate mortar and pestle, and dried in a vacuum oven at 30 °C.

Synthetic speleothem carbonate: A calcite sample (MHD1) was precipitated in a laboratory, under cave-like conditions^55^. Solutions super-saturated relative to calcite were pumped to flow down an inclined, sandblasted glass plate in a thin solution film (0.1 mm in thickness), precipitating CaCO_3_ along the flow path. The experiments were performed in an enclosed space, which allowed control of all surrounding conditions, such as *p*CO_2_, temperature, and relative humidity. Specifically, sample MHD1 was precipitated at 30.7(±0.3) °C, with an atmospheric *p*CO_2_ of 1007(±47) ppm and a relative humidity of 97.5(±1.2)%. The average *δ*^13^C and *δ*^18^O values of the atmospheric CO_2_ were −44.7(±0.8)‰ and −10.6(±0.6)‰ VPDB, respectively. The experimental solution was prepared by dissolving 5 mmol CaCO_3_ powder in high-purity water while bubbling tank CO_2_ through the water column. After the complete dissolution of CaCO_3_ powder, i.e., when there were no visible particles in the solution, the solution was stored for five days at the experimental temperature to obtain isotopic equilibrium among all dissolved inorganic carbon species. This resulted in an initial solution composition of pH = 6.34, [DIC] = 18.19 mM, *δ*^13^C_HCO3−_ ≈ −31.9(±1.3)‰, and *δ*^18^O_HCO3−_ ≈ −8.69(±0.11)‰ VPDB. After being exposed to lower *p*CO_2_ in the climate box, it took ca 18 s for the solution to reach chemical equilibrium with the atmospheric CO_2_, which increased the solution pH and led to super-saturation with respect to calcite. The calcite sample was scratched off the glass plate after the experiment was completed and corresponded to the first 5 cm of flow, i.e., the first 24 s of CaCO_3_ precipitation.

Stalagmite: A calcite sample (SPA121-02) was retrieved from a stalagmite in Spannagel Cave in the Austrian Alps (47.0803 N, 11.6717 E), an extensive cave system with the main entrance at 2523 m above sea level. SPA121-02 is a 4-mm-thin layer within SPA121, a stalagmite that records a long growth history from about 240 to 76 ka. SPA121-02 was formed at about 225 ka during Marine Isotope Stage (MIS) 7.4 when this high-alpine cave was buried beneath a warm-based glacier preventing the cave from freezing^56^. The growth of this stalagmite during MIS 7 likely occurred at constant temperatures around freezing point, i.e., 0(±2) °C. The relatively high *δ*^13^C values of SPA121-02 (about 7‰ VPDB, Supplementary Table 1) was attributed to the disequilibrium isotope effects during peak cold times^56^. A 3 × 6 × 4 mm large piece was cut out from the axial part of the stalagmite SPA121 using a diamond-coated band saw. The piece was then cleaned in an ultrasonic bath in de-ionised water, dried, and crushed and homogenised with an agate mortar and pestle before isotope analysis.

Cold-water coral: A scleractinian, azooxanthellate coral *Desmophyllum pertusum* (formerly known as *Lophelia pertusa*) (JR) was collected alive at Traenadjupet, Norwegian Sea (66.973333 N, 11.108833 E) at a water depth of 300 m during cruise POS325-356/1. The annual mean seawater temperature at the collection location is 7.2(±1.0) °C^57^. With a hand-held drill, a corallite was cut from the axis of the colony, and the septa were removed, i.e., only the theca walls were sampled. The sample was cleaned in an ultrasonic bath using de-ionised water and dried in a vacuum oven at 30 °C before being crushed and homogenised using an agate mortar and pestle.

Warm-water coral: A scleractinian, zooxanthellate coral *Porites lutea* (PC1_2005) was collected at the Rashdoo Atoll, Maldives (4.293776 N, 72.977115 E) at a water depth of ca 1 m. For isotope analysis, a ca 0.5 cm thick section was cut from the coral core. Based on sclerochronological analysis, this section corresponded to the growth in the year 2005 when the annual mean temperature at this location was 29.3(±1.0) °C^58^. The mean annual extension rate of the coral is ca 10 mm year^−1^. To remove material that may have been thermally altered when the section was initially cut from the colony, the surface 0.5 mm was scraped away. Then, the section was cleaned in an ultrasonic bath using de-ionised water and dried in a vacuum oven at 30 °C before being crushed and homogenised using an agate mortar and pestle.

Modern brachiopod shell: A terebratulid brachiopod *Magellania venosa* (Mv143-b) was collected from Punta Gruesa, Chile (42.409833 S, 72.424333 W) from 20 m below sea level. The annual mean temperature at the collection location is 11.4(±1.7) °C^12^. *Magellania venosa* is one of the fastest-growing modern brachiopods, with growth rates ranging from 3.8 mm year^−1^ (adult) to 17.3 mm year^−1^ (juvenile)^59^. For this study, we sampled a ca 2 × 3 cm area in the middle part of the ventral valve. First, we abraded the primary layer of the shell using a slow-speed hand-held drill and a diamond drill bit, cleaned the shell in an ultrasonic bath with de-ionised water, dried it in a vacuum oven at 30 °C, and finally homogenised the material using an agate mortar and pestle. The anterior part of the same specimen showed the largest offset from equilibrium in ∆_47_ values in a previous study^12^.

Cretaceous belemnite: A belemnite *Belemnopsis* sp. (66-4.65) was retrieved from DSDP Site 511 at the Falkland Plateau (51.004667 S, 46.971667 W). The investigated rostrum solidum shows excellent preservation based on cathodoluminescence, and trace element analyses^44,60^. Burial temperatures remained below 100 °C for this core, which makes the solid-state alteration of the clumped isotope composition of this sample unlikely^61,62^. The same sample in this study was analysed for its ∆_47_ values, together with other belemnites, to reconstruct Early Cretaceous southern high latitude palaeotemperatures^44^.

### Mass spectrometry

We performed the CO_2_ clumped isotope analyses of sample carbonates on a Thermo Scientific 253 Plus gas source isotope ratio mass spectrometer during April 2019–March 2020, in three measurement sessions (April–August 2019, September–December 2019, and January–March 2020), following the method of Fiebig et al.^25^. Samples were measured in 5–10 replicates. Each replicate analysis consists of 13 acquisitions (10 cycles of reference and samples comparisons in each acquisition and 20 s integration time during each cycle). The raw clumped isotope values (indicated by subscript “raw” on the ∆ symbol) were calculated using the [Brand]/IUPAC isotopic parameters^29,63^.

### Data correction for the reference carbonates

In order to assign the long-term ∆_47 (CDES90)_ and ∆_48 (CDES90)_ values of the ETH 1, ETH 2, and ETH 3 carbonate reference materials finally used for sample correction (Table 1, see the next section), we followed the correction approach outlined by Fiebig et al.^25^. using equilibrated gases only (subscript “CDES90” on the ∆ symbol indicates that the ∆_47_ and ∆_48_ values of these carbonate reference materials are reported on the Carbon Dioxide Equilibrium Scale at a reaction temperature of 90 °C). A total of 36 aliquots of CO_2_ gases equilibrated at 25 °C and 54 aliquots equilibrated at 1000 °C were considered for the April–August 2019 period (Supplementary Data 1). Data correction for the reference carbonates consisted of two steps: correction for non-linearity followed by correction for scale compression^25,64,65^. These two steps are detailed below.

Correction for non-linearity: Within errors, the two sets of equilibrium gases, equilibrated either at 1000 °C or 25 °C, had identical slopes in ∆_47 (raw)_ vs *δ*_47_ (Supplementary Fig. 3a) and ∆_48 (raw)_ vs *δ*_48_ (Supplementary Fig. 3b) spaces, respectively, when the negative *m/z* 47.5 intensity is directly subtracted from measured *m/z* 47–49 intensities (scaling factor of −1, see below and in Fiebig et al.^25^). We, therefore, considered the slopes displayed by the merged data sets for the correction of non-linearity. In ∆_47 (raw)_ vs *δ*_47_ space, the equilibrium gases showed a flat line such that non-linearity correction needs not be applied. In ∆_48 (raw)_ vs *δ*_48_ space, the slope displayed by the merged data set was −0.0040(±0.0002).

Correction for scale compression: The intercepts for the 1000 °C and the 25 °C gases displayed in ∆_47 (raw)_ vs *δ*_47_ and ∆_48 (raw)_ vs *δ*_48_ spaces were compared to the corresponding theoretical values^66^ to constrain empirical transfer functions (Supplementary Data 1).

Finally, we combined the ∆_47 (CDES90)_ and ∆_48 (CDES90)_ values of ETH 1, ETH 2, and ETH 3 determined during the April–August 2019 period (Supplementary Data 1) with those reported in Fiebig et al.^25^ to calculate the long-term values listed in Table 1 (Supplementary Fig. 4). Shapiro-Wilks tests show that the combined ∆_47 (CDES90)_ and ∆_48 (CDES90)_ data set of the carbonate reference materials have a normal distribution with *W*-values > 0.95 and *p*-values >> 0.05.

### Data correction for the carbonate samples

Unlike the method described in Fiebig et al.^25^, we did not make use of equilibrated gases to correct the samples but used our long-term ∆_47 (CDES90)_ and ∆_48 (CDES90)_ values obtained for ETH 1, ETH 2, and ETH 3 instead (Supplementary Data 2–4). This purely carbonate-based correction approach follows the principle of identical treatment of sample and reference materials and allows identification of subtle temporal drifts in the acid reaction environment and correction for them^67–69^. Correction of the sample data consisted of three steps: correction for non-linearity followed by correction for scale compression, and finally correction for variations in the reaction environment. These three steps are detailed below.

Correction for non-linearity: The negative background causing the non-linearities in ∆_47 (raw)_ vs *δ*_47_, ∆_48 (raw)_ vs *δ*_48_, and ∆_49 (raw)_ vs *δ*_49_ spaces was corrected using Easotope^70^ by subtracting the intensities measured on the *m/z* 47.5 cup from the intensities measured on the *m/z* 47–49 cups, after multiplying the former by respective scaling factors. These scaling factors were determined empirically and enable one to calculate accurate negative backgrounds below *m/z* 47, *m/z* 48, and *m/z* 49 from the measured *m/z* 47.5 intensity. For the three periods of sample analyses, i.e., April–August 2019, September–December 2019, and January–March 2020, we determined the scaling factors in a way that no residual slopes remained between the respective measured values of the frequently analysed ETH 1 and ETH 2 standards in the corresponding ∆ vs *δ* spaces (Supplementary Figs. 5–7). The uniformity of the measured long-term ∆_47 (CDES90)_ and ∆_48 (CDES90)_ values of ETH 1 and ETH 2, also supported by experimental data^71^, allowed us to assume that they have identical ∆_47_ and ∆_48_ values (Table 1). Consequently, for the April–August 2019 period of sample analyses, scaling factors of −0.988, −0.906, and −0.648, respectively, were applied to correct *m/z* 47, *m/z* 48, and *m/z* 49 intensities based on the monitored *m/z* 47.5 intensity. For the September–December 2019 period, the corresponding scaling factors were −1.003, −0.938, and −0.581, respectively. For the January–March 2020 period, factors of −1.010, −0.92326, and −0.555, respectively, were applied.

Correction for scale compression: According to the principles outlined above, we used our long-term ∆_47 (CDES90)_ and ∆_48 (CDES90)_ values of ETH 1, ETH 2, and ETH 3 (Table 1) to project the non-linearity corrected, raw clumped isotope values of the carbonate samples to the CDES. We determined empirical transfer functions based on a comparison of our long-term mean ∆_47 (CDES90)_ and ∆_48 (CDES90)_ values of ETH 1, ETH 2, and ETH 3 (Table 1) with their corresponding, non-linearity corrected ∆_47 (raw)_ and ∆_48 (raw)_ averages over the three periods of sample analysis (Supplementary Figs. 5a–b, 6a–b, 7a–b). The application of these transfer functions to non-linearity corrected sample ∆_47 (raw)_ and ∆_48 (raw)_ values yields the ∆_47 (CDES90,uc)_ and ∆_48 (CDES90,uc)_ values of the samples (Supplementary Data 2–4).

Correction for subtle long-term variations in the acid reaction environment: When residuals between the accepted long-term and the measured ∆ _(CDES90,uc)_ for all ETH 1, ETH 2, and ETH 3 replicate analyses are plotted against time, small but systematic temporal variations become detectable. For ∆_47 (CDES90,uc)_, these residuals are on the order of ≤0.010‰ (Supplementary Figs. 8a, 9a, 10a), and for ∆_48 (CDES90,uc)_ they are on the order of ≤0.030‰ (Supplementary Figs. 8b,  9b, 10b). We determined a residual vs measurement time function (Supplementary Data 2–4) and used it to further correct the ∆_47 (CDES90,uc)_ and ∆_48 (CDES90,uc)_ values in order to obtain the final clumped isotope compositions of the investigated carbonate samples (Table 2).

We used the non-linearity corrected ∆_49 (raw)_ values of the carbonate-derived CO_2_ and the presumably uncontaminated equilibrated CO_2_ gases to check for potential contamination in the analyte. All ∆_49 (raw)_ values of the carbonates fall within the range of the ∆_49 (raw)_ values of the equilibrated gases, indicating no contamination of the investigated solids (Supplementary Figs. 11a–b, 12a–b, 13a–b). In addition, the lack of correlation between ∆_48 (raw)_ and ∆_49 (raw)_ of the measured analytes further argues that there is no contamination on ∆_49_ that would influence ∆_48_ (Supplementary Figs. 11c, 12c, 13c). All measured values can be found in Supplementary Data 1–4.

### Acid fractionation factors

To be able to compare the experimentally measured clumped isotope compositions of a carbonate, i.e., the ∆_47 (CDES90)_ and ∆_48 (CDES90)_ values of the CO_2_ gas derived from the phosphoric acid digestion of that carbonate, with its theoretically predicted composition, i.e., the ∆_63_ and ∆_64_ values of the carbonate, we determined^25^ the clumped isotope fractionation factors associated with the 90 °C acid fractionation during our analysis. These are based on the long-term ∆_47 (CDES90)_ and ∆_48 (CDES90)_ values of ETH 1 and ETH 2 standards which were both potentially equilibrated at 600 °C^68^. The theoretically predicted calcite ∆_63_ and ∆_64_ values at 600 °C are 0.018‰ and 0.002‰^28^, respectively. These, combined with our experimentally measured ∆_47 (CDES90)_ values of 0.212(±0.002)‰ and ∆_48 (CDES90)_ of 0.140(±0.005)‰, yield acid fractionation factors of 0.194(±0.002)‰ for ∆_63_–∆_47_ and 0.138(±0.005)‰ for ∆_64_–∆_48_.

### Numerical modelling

We used numerical models to simulate the evolution of the isotopic composition of the DIC during (1) CO_2_ absorption, i.e., the key process involved in coral calcification^26^, and (2) the laboratory carbonate precipitation of the synthetic speleothem^27^ (Supplementary Data 5).

(1) CO_2_ absorption simulations were constructed using the IsoDIC model to mimic the internal calcification environment of scleractinian corals^26^. Specifically, the modelled calcification environment consisted of an aqueous solution ([DIC] = 2 mM, *δ*^13^C_DIC_ = 0, and pH = 8.8 for cold-water corals and pH = 8.5 for warm-water corals), which was exposed to a CO_2_-containing atmosphere (*p*CO_2_ = 1100 ppm^72^ and *δ*^13^C_CO2_ = −15‰^73^). The temperature of the modelled calcification environment corresponded to the mean growth temperatures of the cold- and warm-water corals, i.e., 7.2 °C and 28.9 °C, respectively. The catalytic enhancement of the inter-conversion between CO_2 (aq)_ and HCO_3_^−^ by carbonic anhydrase during coral calcification is simulated by increasing the rate constants of CO_2 (aq)_ (de)hydration reactions^26^. The initial oxygen and clumped isotope compositions of both the DIC and air CO_2_ were assumed to be in isotopic equilibrium with the water (*δ*^18^O_H2O_ = 0 VSMOW) at the above described temperatures.

(2) To model the isotopic composition of the synthetic speleothem, simulations were constructed using the IsoCave model^27^, based on the conditions of the laboratory experiment^55^ (*T* = 30.7 °C, *p*CO_2_ = 1007 ppm, water film thickness of 100 μm, *δ*^13^C_CO2_ = −44.7‰, *δ*^18^O_CO2_ = −10.6‰ VPDB, *δ*^13^C_CaCO3_ = −6‰, *δ*^18^O_H2O_ = −9‰ VSMOW, see above as well) and yielded an initial solution composition of pH = 6.3, [DIC] = 18.1 mM, [Ca^2+^] = 4.9 mM, *δ*^13^C_HCO3−_ = −31.2‰, and *δ*^18^O_HCO3−_ = −9.0‰ VPDB, which are close to the experimentally determined values (pH = 6.34, [DIC] = 18.2 mM, [Ca^2+^] = 5 mM, *δ*^13^C_HCO3−_ ≈ −31.9(±1.3)‰, and *δ*^18^O_HCO3−_ ≈ −8.69(±0.11)‰ VPDB, see above).

## Supplementary information

Supplementary Information

Description of Additional Supplementary Files

Supplementary Data 1

Supplementary Data 2

Supplementary Data 3

Supplementary Data 4

Supplementary Data 5

## Data Availability

The analytical and model data supporting the findings of this study are available within the article, its Supplementary Information, and Supplementary Data files. All data files are additionally deposited at: [10.5281/zenodo.3784963]

## Code availability

The figures were generated using code written in R, available at: [10.5281/zenodo.3784963]
