# Supplementary material for: Dual clumped isotope thermometry resolves kinetic biases in carbonate formation temperatures
Source: Nat Commun. 2020 Aug 10;11:4005. doi: 10.1038/s41467-020-17501-0 (PMC7418028; doi:10.1038/s41467-020-17501-0)
Supplement: Supplementary file 1 — Supplementary Information [file 41467_2020_17501_MOESM1_ESM.pdf]

Supplementary Information for

Dual clumped isotope thermometry resolves kinetic biases in carbonate formation temperatures

by Bajnai et al.

Supplementary Table 1 Stable isotope composition of the samples and their parent waters

Sample (type)	$\delta^{13}C$ carbonate (‰ VPDB)	$\delta^{18}O$ carbonate (‰ VSMOW)	$\delta^{18}O$ water (‰ VSMOW)
DHC2-8 (vein calcite)	-1.92 (±0.02)	14.49 (±0.03)	-13.54 (±0.05) (from Coplen[1])
MSK 2b (cryogenic cave carbonate)	7.63 (±0.02)	12.10 (±0.10)	–
Obi 87-i (pool carbonate)	-8.64 (±0.04)	22.81 (±0.05)	–
MHD1 (synthetic speleothem)	-33.53 (±0.11)	20.08 (±0.06)	-9.47 (±0.10) (from Hansen *et al.*[2])
SPA121-02 (stalagmite)	7.62 (±0.03)	17.66 (±0.05)	–
Mv143-b (brachiopod)	-0.29 (±0.03)	30.91 (±0.04)	-0.31 (from Schmidt *et al.*[3], GSO18Db)
66-4.65 (belemnite)	0.16 (±0.02)	29.59 (±0.05)	–
JR (cold-water coral)	-7.84 (±0.09)	30.13 (±0.08)	0.49 (from Schmidt *et al.*[3], GSO18Db)
PC1_2005 (warm-water coral)	-1.59 (±0.04)	25.38 (±0.10)	0.52 (±0.12) (from Storz *et al.*[4])

The ± uncertainties reflect external 2 standard errors that include the *t*-value. The isotopic compositions of the carbonate derived CO_2 gases were measured against a CO_2 reference gas (ISO-TOP, Air Liquide) with a $\delta^{18}O$ value of 25.26‰ VSMOW and $\delta^{13}C$ value of -4.20‰ VPDB. Carbonate $\delta^{18}O$ values consider the 90 °C acid fractionation factors between carbonate (calcite or aragonite) and CO_2 of Kim *et al.*[5].

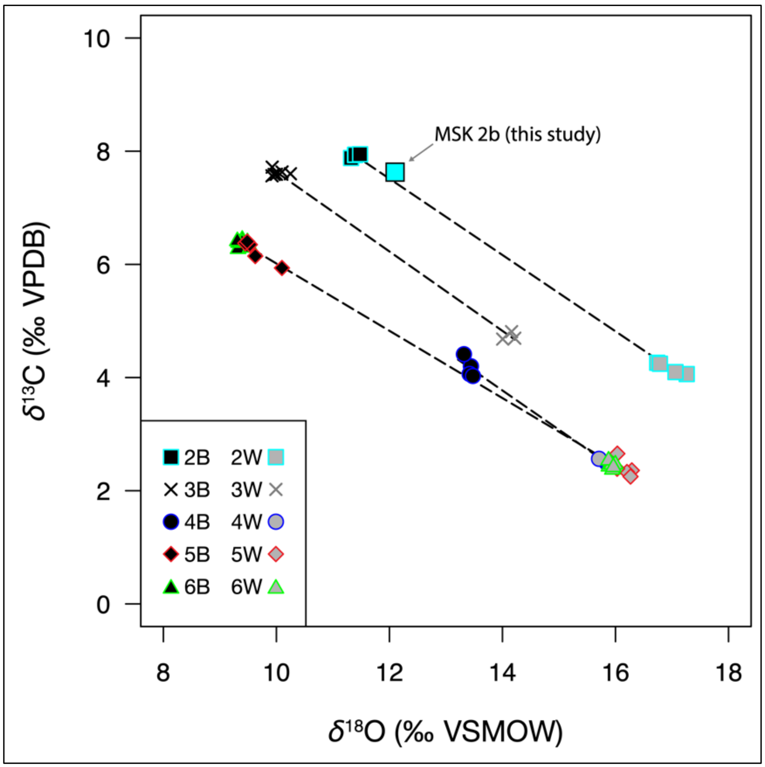

Supplementary Figure 1 | Our cryogenic cave carbonate sample likely crystallised close to isotopic equilibrium. All cryogenic cave carbonate samples from the cave where our MSK 2b sample was collected (see Methods) show crystal morphologies as well as $\delta^{13}C$ and $\delta^{18}O$ values that are diagnostic of coarsely crystalline cryogenic cave carbonates[6]. Data of individual crystals and aggregates thereof plot along straight lines representing different stages of progressive freezing (dashed lines). The $\delta^{13}C$ and $\delta^{18}O$ values of MSK 2b (cyan square; see Supplementary Table 1) is typical of the final stage of the freezing process, whereby a meltwater pool enclosed in cave ice is progressively converted to ice. Ice sheet growth, in turn, progressively inhibits carbon dioxide degassing. As a consequence, calcium carbonate precipitation slows down[7], such that the late-stage cryogenic crystal MSK 2b likely crystallised close to isotopic equilibrium at around 0(±2) °C. Clumped isotope data of coarsely crystalline cryogenic cave carbonates from various caves in Germany support this notion by showing that those samples with the highest $\delta^{13}C$ and the lowest $\delta^{18}O$ values, i.e., the late-stage precipitates, attain Δ_{47} values close to equilibrium[8]. Figure adapted from Spötl and Cheng[6].

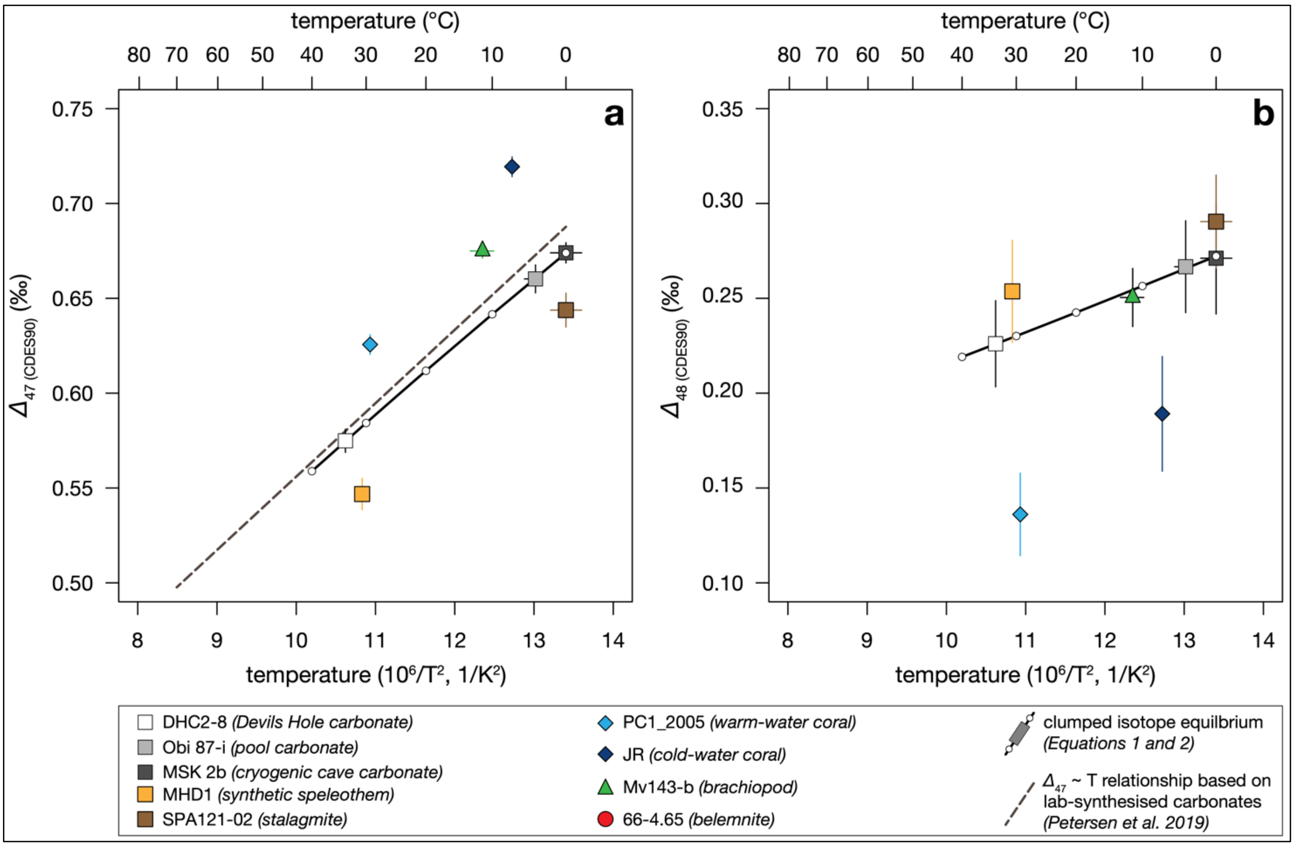

Supplementary Figure 2 | The temperature dependence of clumped isotope equilibrium.

(**a**) The $\Delta_{47\,(CDES90)}$ values of the samples plotted against their formation temperature. The black line depicts the equilibrium temperature dependence of $\Delta_{47\,(CDES90)}$ described in the main text, i.e., Equation 1. The dashed line depicts the Δ_{47} vs temperature relationship derived from laboratory-synthesised carbonates[9], i.e., $\Delta_{47\,(CDES90)} = 0.0387(\pm1.7\text{x}10^{-6}) \text{ x } 10^6/T^2 + 0.169(1.7\text{x}10^{-5})$ (**b**) The $\Delta_{48\,(CDES90)}$ values of the samples plotted against their formation temperature. The black line depicts the equilibrium temperature dependence of $\Delta_{48\,(CDES90)}$, i.e., Equation 2 in the main text. Subscript "CDES90" on the Δ symbol indicates that the Δ_{47} and Δ_{48} values of these carbonates are reported on the Carbon Dioxide Equilibrium Scale at a reaction temperature of 90 °C. All error bars depict 2 standard errors (95% confidence interval[10]).

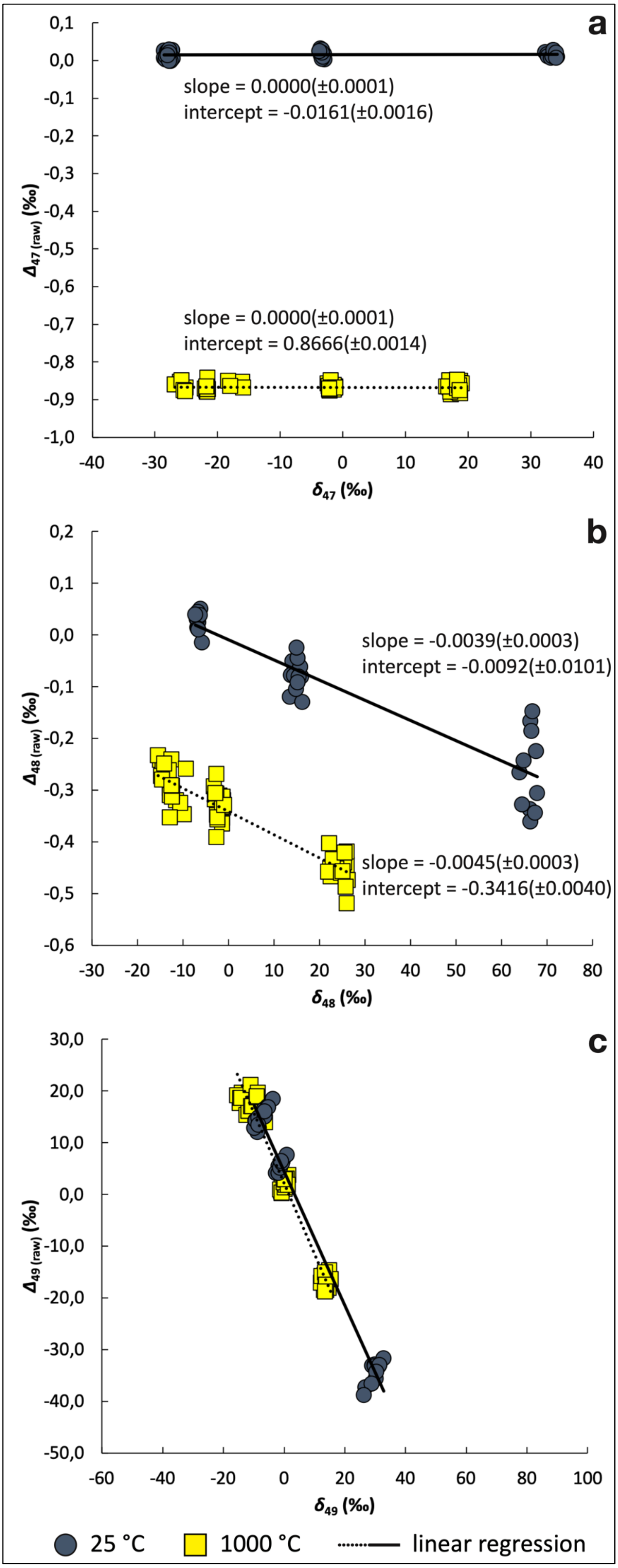

Supplementary Figure 3 | Raw isotope data of equilibrium gases from the April–August 2019 measurement period.

The raw isotope values are calculated with a uniform *m/z* 47.5 scaling factor of -1 for *m/z* 47–49. (Supplementary Data 1). (**a**) Slopes and intercepts, and the corresponding errors of the 1000 °C and 25 °C equilibrated CO_2 gases in $\Delta_{47\ (raw)}$ vs δ_{47} space. (**b**) Slopes and intercepts, and the corresponding errors of the 1000 °C and 25 °C equilibrated CO_2 gases in $\Delta_{48\ (raw)}$ vs δ_{48} space. (**c**) 1000 °C and 25 °C equilibrated CO_2 gases in $\Delta_{49\ (raw)}$ vs δ_{49} space.

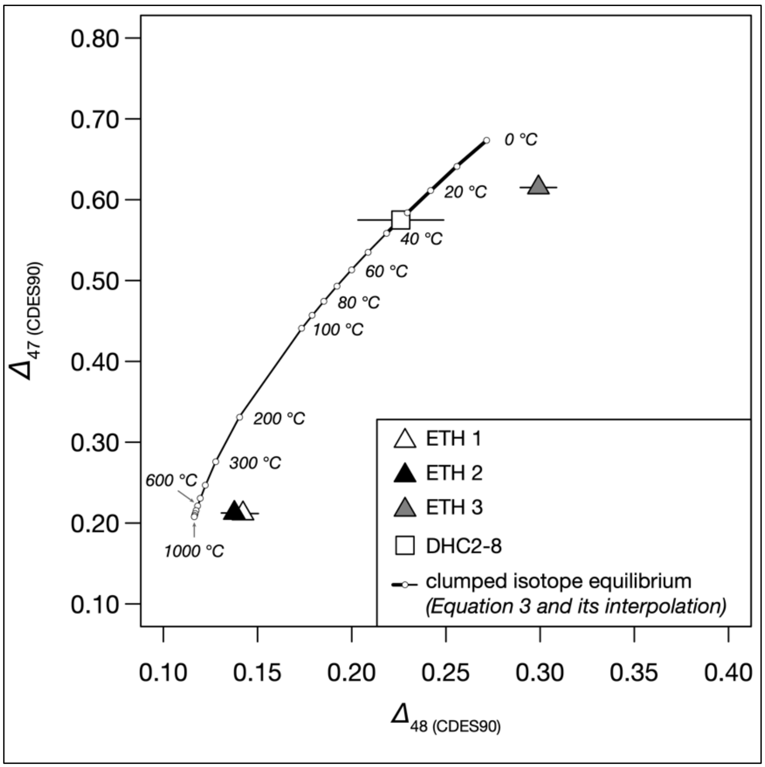

Supplementary Figure 4 | Carbonate reference materials in $\Delta_{47\ (CDES90)}$ vs $\Delta_{48\ (CDES90)}$ space.

This plot shows long-term data for ETH 1, ETH 2, and ETH 3 carbonate reference materials from the April–August 2019 measurement period combined with values reported in Fiebig *et al.*[11]. The corresponding $\Delta_{47\ (CDES90)}$ and $\Delta_{48\ (CDES90)}$ values of the ETH reference materials are reported in Table 1. Equation 3, valid in the 0–40 °C temperature range, was extrapolated to high temperatures assuming the theoretical temperature sensitivity of Hill *et al.*[12] and the differences between the $\Delta_{47\ (CDES90)}$ and $\Delta_{48\ (CDES90)}$ values of DHC2-8 and the corresponding values predicted by our empirical relationships at 33.7 °C (see Results) are constant over the entire temperature range. The apparent deviation of ETH 3, a Cretaceous chalk, from our equilibrium Δ_{47} vs Δ_{48} line may be explained by diagenesis at a temperature lower than its formation temperature. The apparent deviation of ETH 1 and ETH 2 (carbonates heated to 600 °C for 10 h and quenched to room temperature during 40 min[(13)]) from our equilibrium line could be due to partial resetting during quenching. Alternatively, the deviations for ETH 1 and ETH 2 could derive from the uncertainties with our current equilibrium line at high temperatures. For example, the differences between the Δ values obtained for DHC2-8 and the corresponding values predicted by our empirical relationships, may not be constant over the entire temperature range (0–1000 °C).

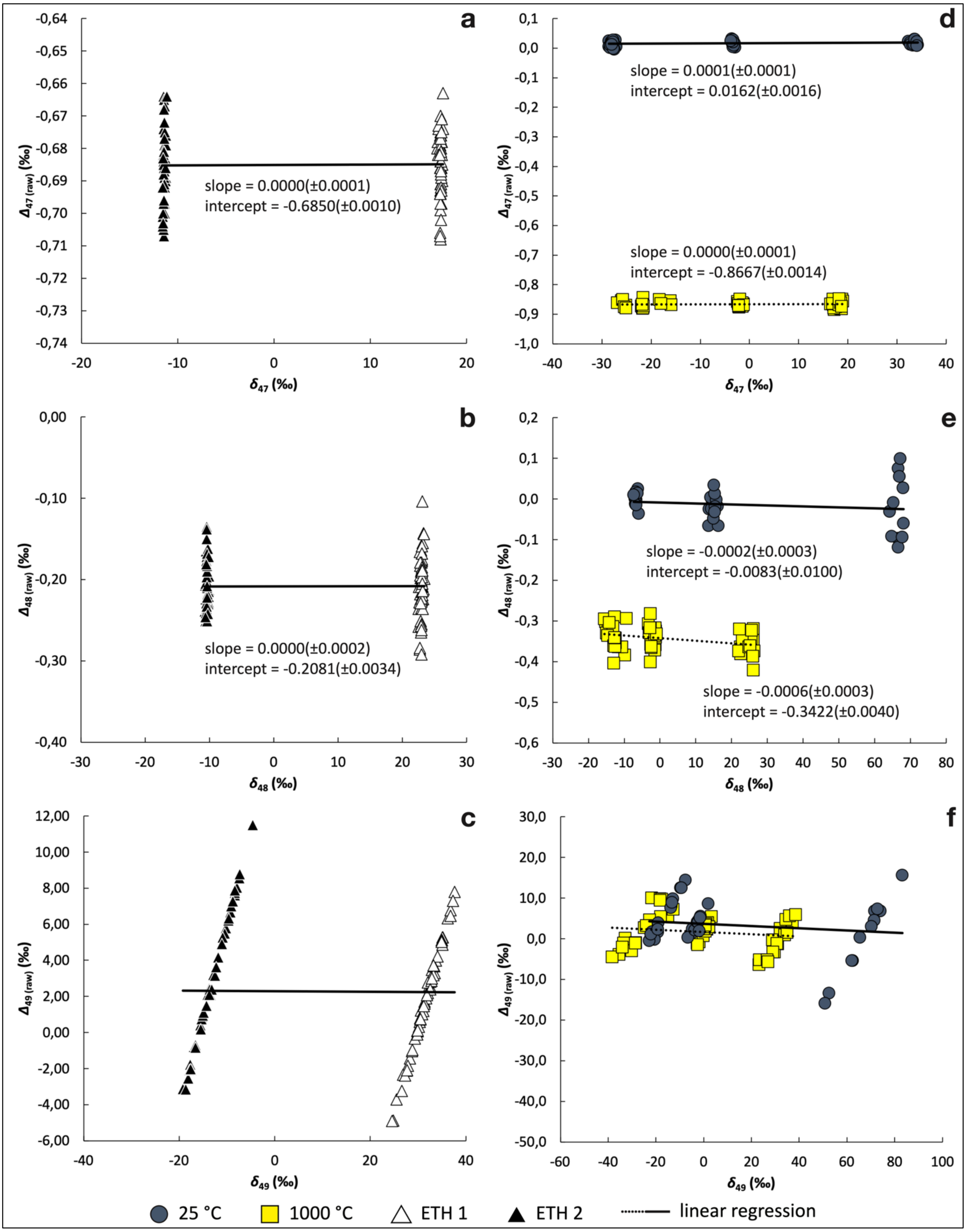

a
slope = 0.0000(±0.0001)
intercept = -0.6850(±0.0010)
Δ47 (raw) (‰)
δ47 (‰)
b
slope = 0.0000(±0.0002)
intercept = -0.2081(±0.0034)
Δ48 (raw) (‰)
δ48 (‰)
c
Δ49 (raw) (‰)
δ49 (‰)
d
slope = 0.0001(±0.0001)
intercept = 0.0162(±0.0016)
slope = 0.0000(±0.0001)
intercept = -0.8667(±0.0014)
Δ47 (raw) (‰)
δ47 (‰)
e
slope = -0.0002(±0.0003)
intercept = -0.0083(±0.0100)
slope = -0.0006(±0.0003)
intercept = -0.3422(±0.0040)
Δ48 (raw) (‰)
δ48 (‰)
f
Δ49 (raw) (‰)
δ49 (‰)
25 °C
1000 °C
ETH 1
ETH 2
linear regression

Supplementary Figure 5 (on the previous page) | Non-linearity corrected raw isotope data of equilibrium gases and carbonate standards from the April–August 2019 measurement period. The raw isotope values were calculated with empirically derived scaling factors of -0.988, -0.906, and -0.648 for *m/z* 47–49, respectively (see Methods; Supplementary Data 2). We chose the *m/z* 47.5 intensity scaling factors for the background correction in a way that no residual slopes remain between the respective measured δ and Δ values of the ETH 1 and ETH 2 standards, i.e., we adjusted the scaling factors in Easotope until we got a 0 slope between ETH 1 and ETH 2. (**a,d**) The slope in $\Delta_{47\,(raw)}$ vs δ_{47} space between ETH 1 and ETH 2, and the 25 °C and the 1000 °C gases, respectively, are indistinguishable from zero. (**b,e**) In $\Delta_{48\,(raw)}$ vs δ_{48} space, there is no residual slope between ETH 1 and ETH 2. The residual slopes between the 25 °C and the 1000 °C CO_2 gases are indistinguishable from each other. The residual slope of the merged 25 °C and 1000 °C datasets (-0.0003±0.0002, Supplementary Data 2) is indistinguishable from the residual slope between ETH 1 and ETH 2 (0.0000±0.0002). (**c,f**) Raw isotope values of ETH 1 and ETH 2, and the equilibrated gases in $\Delta_{49\,(raw)}$ vs δ_{49} space.

Note, that the $\Delta_{47\,(raw)}$ and $\Delta_{48\,(raw)}$ values of the 1000 °C and 25 °C equilibrated CO_2 gases (**d,e**) were not directly used in the calculation of the $\Delta_{47\,(CDES90)}$ and $\Delta_{48\,(CDES90)}$ values of the samples. Instead, the $\Delta_{47\,(CDES90)}$ and $\Delta_{48\,(CDES90)}$ values of the samples were determined based on the long-term $\Delta_{47\,(CDES90)}$ and $\Delta_{48\,(CDES90)}$ values of the ETH 1, ETH 2, and ETH 3 reference carbonates (Table 1; see Methods).

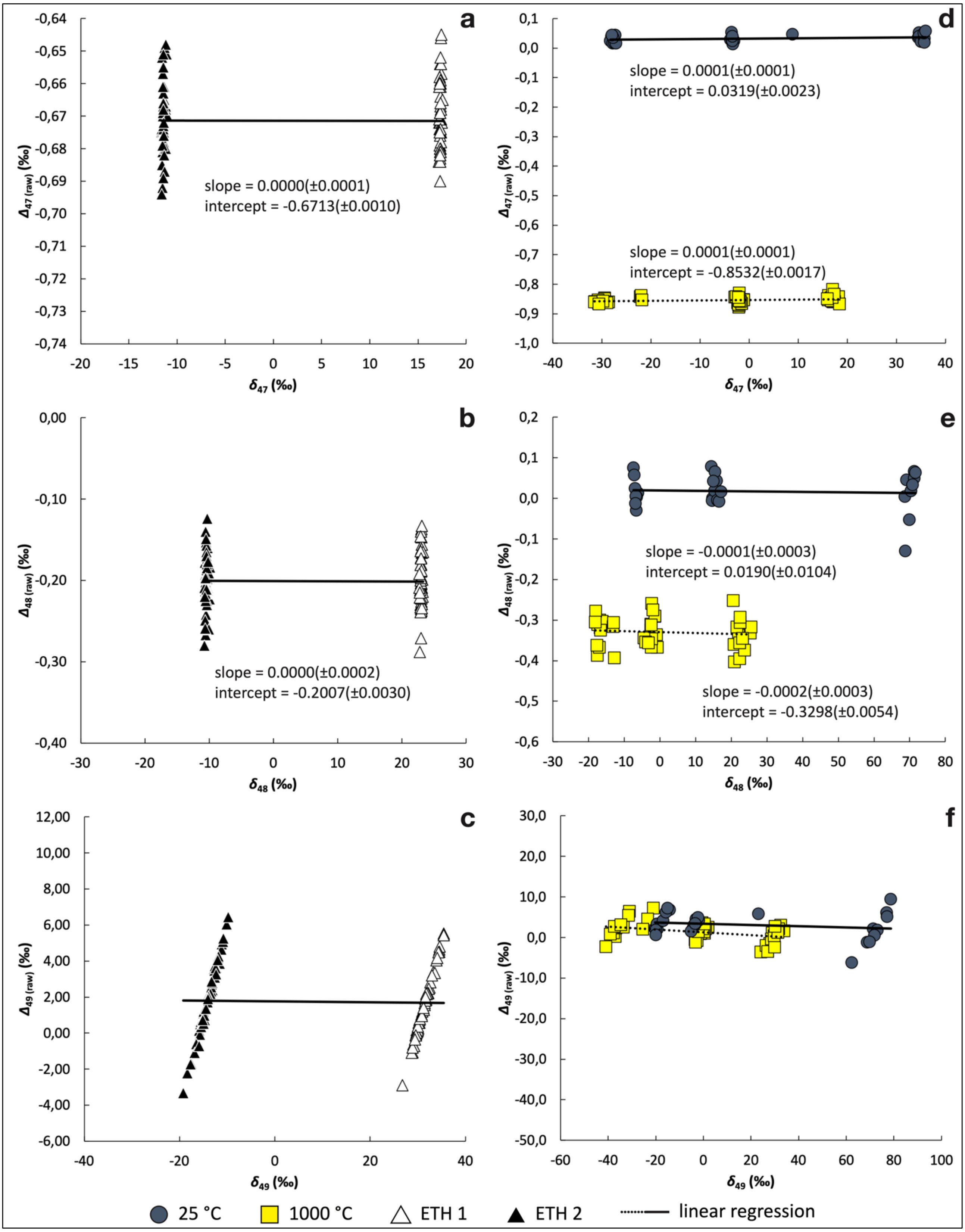
a
slope = 0.0000(±0.0001)
intercept = -0.6713(±0.0010)
d
slope = 0.0001(±0.0001)
intercept = 0.0319(±0.0023)
slope = 0.0001(±0.0001)
intercept = -0.8532(±0.0017)
b
slope = 0.0000(±0.0002)
intercept = -0.2007(±0.0030)
e
slope = -0.0001(±0.0003)
intercept = 0.0190(±0.0104)
slope = -0.0002(±0.0003)
intercept = -0.3298(±0.0054)
c
f
25 °C
1000 °C
ETH 1
ETH 2
linear regression

Supplementary Figure 6 (on the previous page) | Non-linearity corrected raw isotope data of equilibrium gases and carbonate standards from the Sept–Dec 2019 measurement period. The raw isotope values were calculated with empirically derived scaling factors of -1.003, -0.938, and -0.581 for *m/z* 47–49, respectively (see Methods; Supplementary Data 3). We chose the *m/z* 47.5 intensity scaling factors for the background correction in a way that no residual slopes remain between the respective measured δ and Δ values of the ETH 1 and ETH 2 standards, i.e., we adjusted the scaling factors in Easotope until we got a 0 slope between ETH 1 and ETH 2. (**a,d**) The slope in $\Delta_{47\ (raw)}$ vs δ_{47} space between ETH 1 and ETH 2, and the 25 °C and the 1000 °C gases, respectively, are indistinguishable from zero. (**b,e**) The slope in $\Delta_{48\ (raw)}$ vs δ_{48} space between ETH 1 and ETH 2, and the 25 °C and the 1000 °C gases, respectively, are indistinguishable from zero. (**c,f**) Raw isotope values of ETH 1 and ETH 2, and the equilibrated gases in $\Delta_{49\ (raw)}$ vs δ_{49} space.

Note, that the $\Delta_{47\ (raw)}$ and $\Delta_{48\ (raw)}$ values of the 1000 °C and 25 °C equilibrated CO_2 gases (**d,e**) were not directly used in the calculation of the $\Delta_{47\ (CDES90)}$ and $\Delta_{48\ (CDES90)}$ values of the samples. Instead, the $\Delta_{47\ (CDES90)}$ and $\Delta_{48\ (CDES90)}$ values of the samples were determined based on the long-term $\Delta_{47\ (CDES90)}$ and $\Delta_{48\ (CDES90)}$ values of the ETH 1, ETH 2, and ETH 3 reference carbonates (Table 1; see Methods).

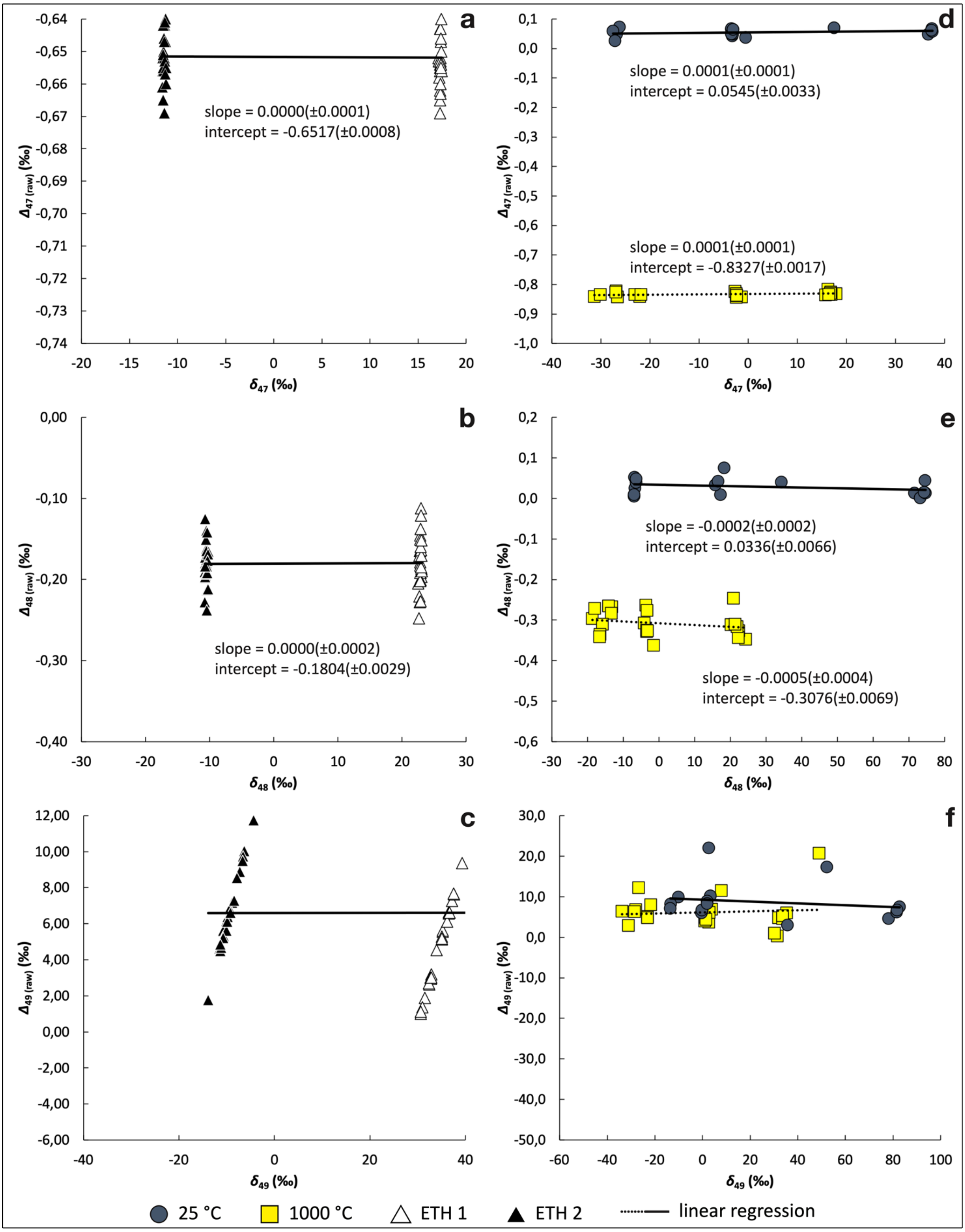
a
slope = 0.0000(±0.0001)
intercept = -0.6517(±0.0008)
$\Delta_{47\,(raw)}$ (‰)
δ_{47} (‰)
d
slope = 0.0001(±0.0001)
intercept = 0.0545(±0.0033)
slope = 0.0001(±0.0001)
intercept = -0.8327(±0.0017)
$\Delta_{47\,(raw)}$ (‰)
δ_{47} (‰)
b
slope = 0.0000(±0.0002)
intercept = -0.1804(±0.0029)
$\Delta_{48\,(raw)}$ (‰)
δ_{48} (‰)
e
slope = -0.0002(±0.0002)
intercept = 0.0336(±0.0066)
slope = -0.0005(±0.0004)
intercept = -0.3076(±0.0069)
$\Delta_{48\,(raw)}$ (‰)
δ_{48} (‰)
c
$\Delta_{49\,(raw)}$ (‰)
δ_{49} (‰)
f
$\Delta_{49\,(raw)}$ (‰)
δ_{49} (‰)
25 °C
1000 °C
ETH 1
ETH 2
linear regression

Supplementary Figure 7 (on the previous page) | Non-linearity corrected raw isotope data of equilibrium gases and carbonate standards from the January–March 2020 measurement period. The raw isotope values were calculated with empirically derived scaling factors of -1.010, -0.92326, and -0.555 for *m*/*z* 47–49, respectively (see Methods; Supplementary Data 4). We chose the *m*/*z* 47.5 intensity scaling factors for the background correction in a way that no residual slopes remain between the respective measured *δ* and *Δ* values of the ETH 1 and ETH 2 standards, i.e., we adjusted the scaling factors in Easotope until we got a 0 slope between ETH 1 and ETH 2. (**a,d**) The slope in $\Delta_{47\,(\mathrm{raw})}$ vs δ_{47} space between ETH 1 and ETH 2, and the 25 °C and the 1000 °C gases, respectively, are indistinguishable from zero. (**b,e**) In $\Delta_{48\,(\mathrm{raw})}$ vs δ_{48} space, there is no residual slope between ETH 1 and ETH 2. The residual slopes between the 25 °C and the 1000 °C CO_2 gases are indistinguishable from each other. The residual slope of the merged 25 °C and 1000 °C datasets (-0.0002±0.0002, Supplementary Data 4) is indistinguishable from the residual slope between ETH 1 and ETH 2 (0.0000±0.0002). (**c,f**) Raw isotope values of ETH 1 and ETH 2, and the equilibrated gases in $\Delta_{49\,(\mathrm{raw})}$ vs δ_{49} space.

Note, that the $\Delta_{47\,(\mathrm{raw})}$ and $\Delta_{48\,(\mathrm{raw})}$ values of the 1000 °C and 25 °C equilibrated CO_2 gases (**d,e**) were not directly used in the calculation of the $\Delta_{47\,(\mathrm{CDES90})}$ and $\Delta_{48\,(\mathrm{CDES90})}$ values of the samples. Instead, the $\Delta_{47\,(\mathrm{CDES90})}$ and $\Delta_{48\,(\mathrm{CDES90})}$ values of the samples were determined based on the long-term $\Delta_{47\,(\mathrm{CDES90})}$ and $\Delta_{48\,(\mathrm{CDES90})}$ values of the ETH 1, ETH 2, and ETH 3 reference carbonates (Table 1; see Methods).

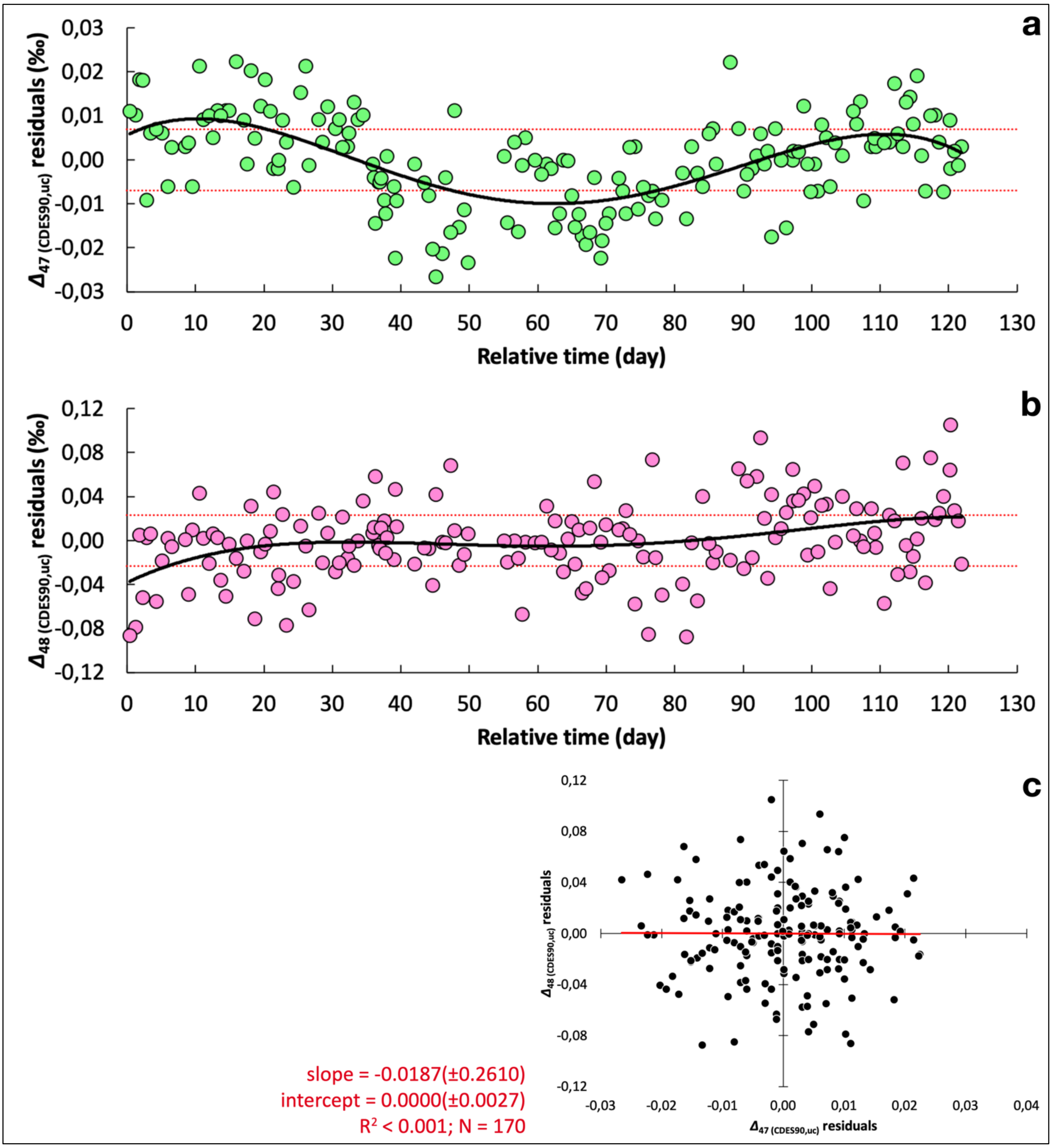

Supplementary Figure 8 | Variations in the acid digestion environment (April–August 2019).

This plot shows data from the April–August 2019 measurement period (Supplementary Data 2). The relative time axis displays the difference in days since 2019-04-05 00:01 CEST. Residual is the difference between the $\Delta_{(CDES90,uc)}$ values of ETH 1, ETH 2, and ETH 3 and the corresponding long-term $\Delta_{(CDES90)}$ values (Table 1). The dotted red lines indicate the shot-noise range of a single replicate measurement[14]. (**a**) A 4th order polynomial was fitted to the $\Delta_{47\ (CDES90,uc)}$ residuals to correct for systematic temporal variations. (**b**) A 4th order polynomial was fitted to the $\Delta_{48\ (CDES90,uc)}$ residuals to correct for systematic temporal variations. (**c**) There is no correlation between $\Delta_{47\ (CDES90,uc)}$ and $\Delta_{48\ (CDES90,uc)}$ residuals. The mechanism that causes deviations from the long-term $\Delta_{47\ (CDES90)}$ value could not be resolved in the $\Delta_{48\ (CDES90,uc)}$ values.

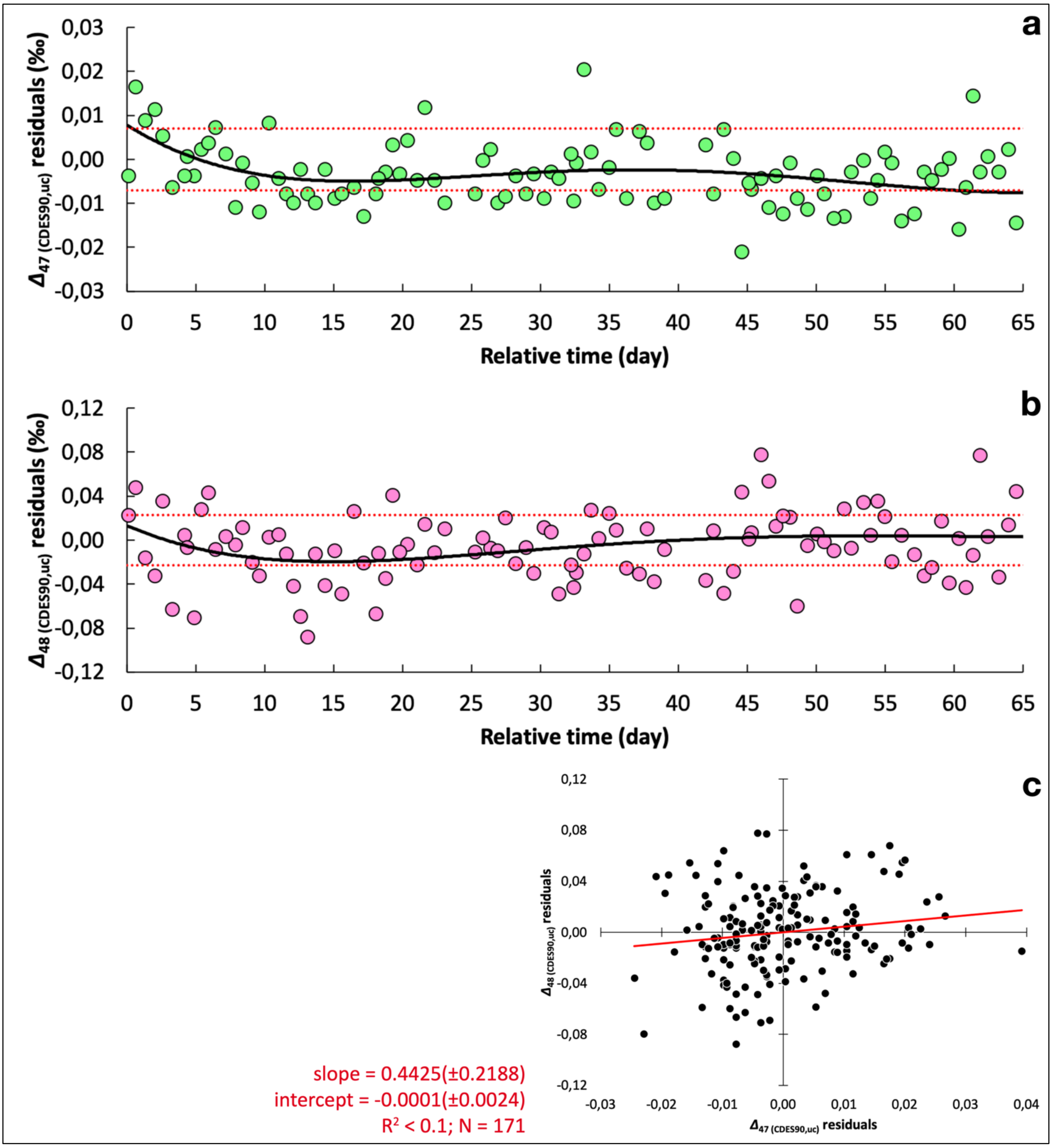

Supplementary Figure 9 | Variations in the acid digestion environment (Sept–Dec 2019).

This plot shows data from the September–December 2019 measurement period (Supplementary Data 3). The relative time axis displays the difference in days since 2019-09-03 00:01 CEST. Residual is the difference between the $\Delta_{(CDES90,uc)}$ values of the ETH 1, ETH 2, and ETH 3 standards and the corresponding long-term $\Delta_{(CDES90)}$ values (Table 1). The dotted red lines indicate the shot-noise range of a single replicate measurement[14]. (**a**) A 6th order polynomial was fitted to the $\Delta_{47\ (CDES90,uc)}$ residuals to correct for systematic temporal variations. (**b**) A 6th order polynomial was fitted to the $\Delta_{48\ (CDES90,uc)}$ residuals to correct for systematic temporal variations. (**c**) There is only a minor correlation between $\Delta_{47\ (CDES90,uc)}$ and $\Delta_{48\ (CDES90,uc)}$ residuals.

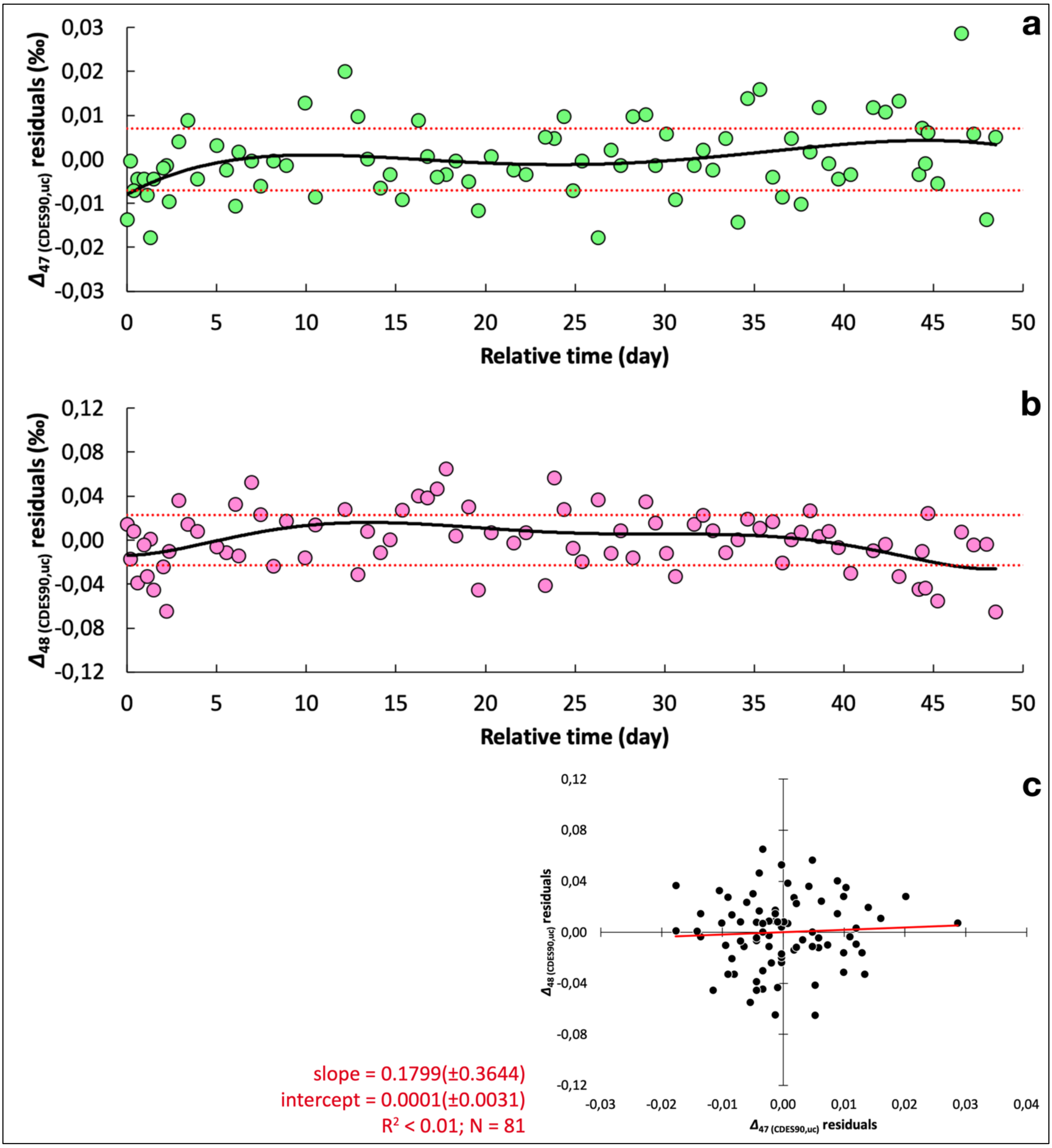

Supplementary Figure 10| Variations in the acid digestion environment (January–March 2020). This plot shows data from the January–March 2020 measurement period (Supplementary Data 4). The relative time axis displays the difference in days since 2020-01-16 00:01 CET. Residual is the difference between the $\Delta_{(CDES90,uc)}$ values of the ETH 1, ETH 2, and ETH 3 standards and the corresponding long-term $\Delta_{(CDES90)}$ values (Table 1). The dotted red lines indicate the shot-noise range of a single replicate measurement[14]. (**a**) A 5th order polynomial was fitted to the $\Delta_{47\,(CDES90,uc)}$ residuals to correct for systematic temporal variations. (**b**) A 6th order polynomial was fitted to the $\Delta_{48\,(CDES90,uc)}$ residuals to correct for systematic temporal variations. (**c**) There is only a minor correlation between $\Delta_{47\,(CDES90,uc)}$ and $\Delta_{48\,(CDES90,uc)}$ residuals.

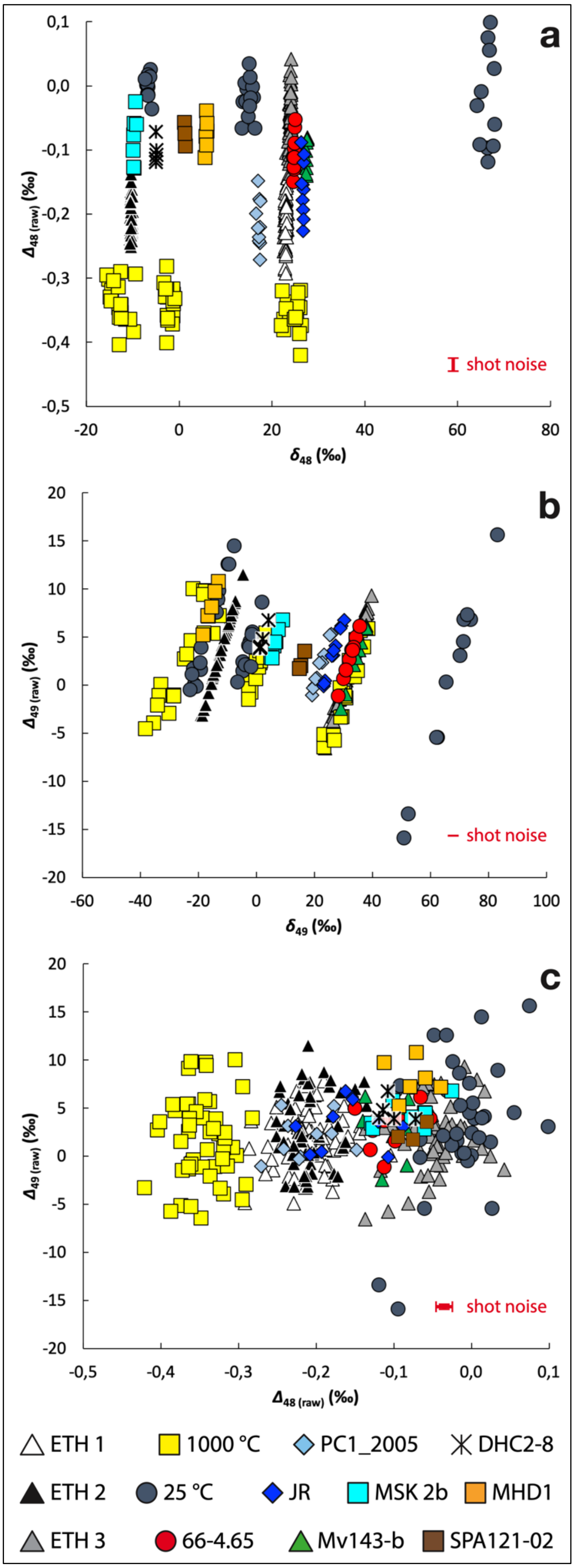

Supplementary Figure 11 | No evidence for contamination in the carbonate-derived CO_2 gases (April–August 2019). This plot shows non-linearity corrected raw data from the April–August 2019 measurement period. The raw isotope values were calculated with the empirically derived scaling factors (see Methods; Supplementary Data 2, Supplementary Figure 5). (**a**) The $\Delta_{48\,(raw)}$ values of the carbonate-derived CO_2 plot between the $\Delta_{48\,(raw)}$ values of the presumably uncontaminated equilibrated CO_2 gases. (**b**) The $\Delta_{49\,(raw)}$ values of the carbonate-derived CO_2 plot between the $\Delta_{49\,(raw)}$ values of the presumably uncontaminated equilibrated CO_2 gases. (**c**) $\Delta_{49\,(raw)}$ vs $\Delta_{48\,(raw)}$. The shot noise limit for Δ_{48} and Δ_{49} are 0.023‰ and 0.224‰, respectively[14].

All $\Delta_{49\,(raw)}$ values of the carbonates fall within the range of the $\Delta_{49\,(raw)}$ values of the equilibrated gases, indicating no additional contamination of the investigated solids relative to the equilibrated gases. The absolute scatter of replicate data is clearly higher than the corresponding shot noise limit. However, for each sample, there is a lack of correlation between $\Delta_{48\,(raw)}$ and $\Delta_{49\,(raw)}$ values, demonstrating that, whatever evokes the scatter in $\Delta_{49\,(raw)}$ values, has not affected $\Delta_{48\,(raw)}$ values.

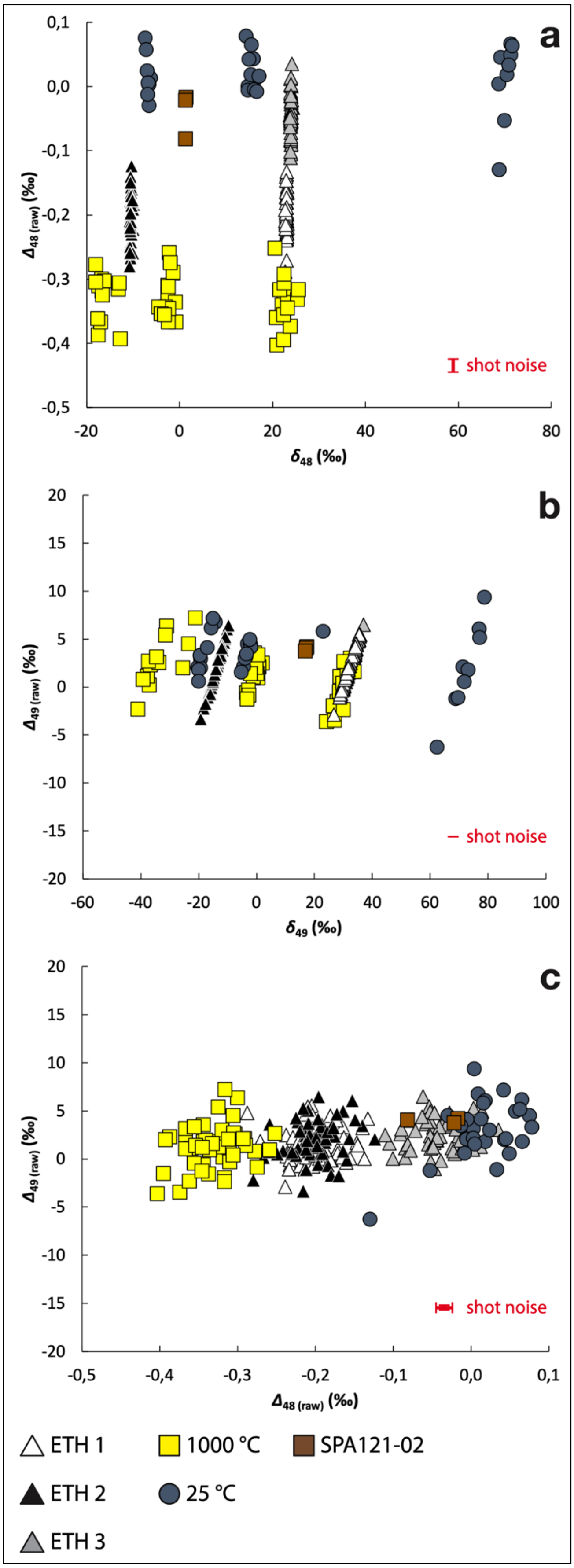

Supplementary Figure 12 | No evidence for contamination in the carbonate-derived CO_2 gases (September–December 2019). This plot shows non-linearity corrected raw data from the September–December 2019 measurement period. The raw isotope values were calculated with the empirically derived scaling factors (see Methods; Supplementary Data 3, Supplementary Figure 6). (**a**) The $\Delta_{48\,(raw)}$ values of the carbonate-derived CO_2 plot between the $\Delta_{48\,(raw)}$ values of the presumably uncontaminated equilibrated CO_2 gases. (**b**) The $\Delta_{49\,(raw)}$ values of the carbonate-derived CO_2 plot between the $\Delta_{49\,(raw)}$ values of the presumably uncontaminated equilibrated CO_2 gases. (**c**) $\Delta_{49\,(raw)}$ vs $\Delta_{48\,(raw)}$. The shot noise limit for Δ_{48} and Δ_{49} are 0.023‰ and 0.224‰, respectively[14].

All $\Delta_{49\,(raw)}$ values of the carbonates fall within the range of the $\Delta_{49\,(raw)}$ values of the equilibrated gases, indicating no additional contamination of the investigated solids relative to the equilibrated gases. The absolute scatter of replicate data is clearly higher than the corresponding shot noise limit. However, for each sample, there is a lack of correlation between $\Delta_{48\,(raw)}$ and $\Delta_{49\,(raw)}$ values, demonstrating that, whatever evokes the scatter in $\Delta_{49\,(raw)}$ values, has not affected $\Delta_{48\,(raw)}$ values.

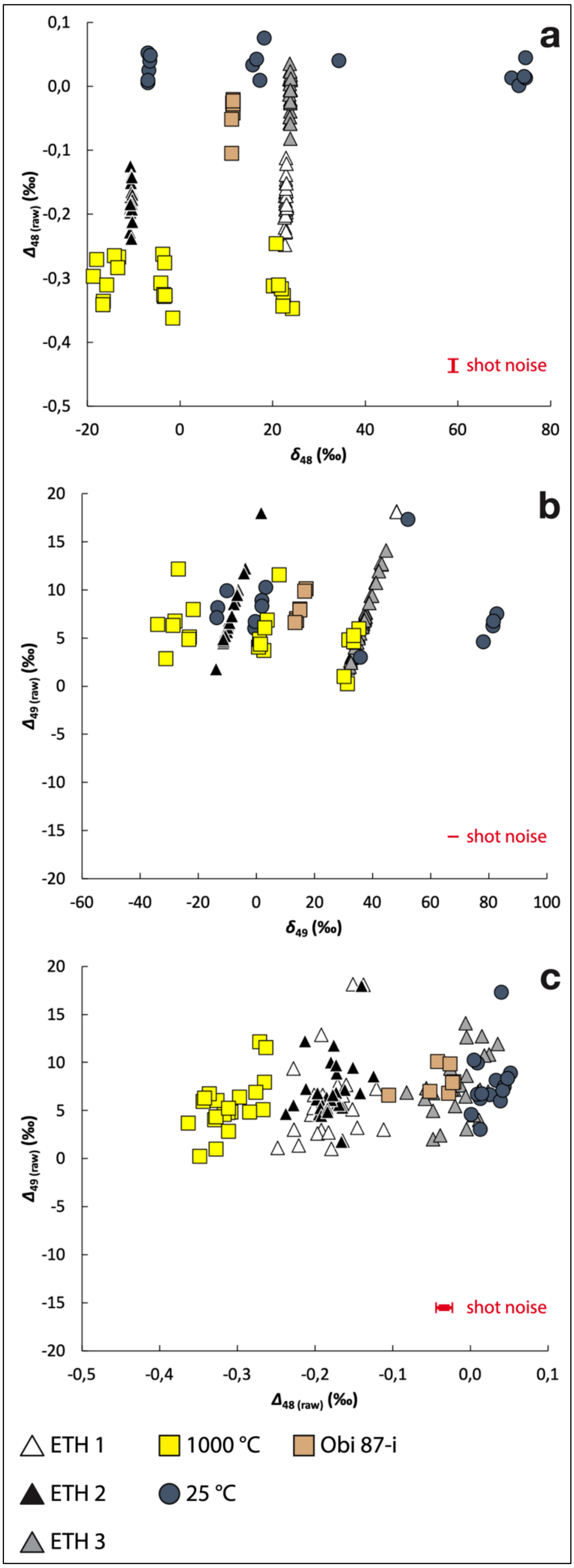

Supplementary Figure 13 | No evidence for contamination in the carbonate-derived CO_2 gases (January–March 2020).

This plot shows non-linearity corrected raw data from the January–March 2020 measurement period. The raw isotope values were calculated with the empirically derived scaling factors (see Methods; Supplementary Data 4, Supplementary Figure 7). (**a**) The $\Delta_{48\,(raw)}$ values of the carbonate-derived CO_2 plot between the $\Delta_{48\,(raw)}$ values of the presumably uncontaminated equilibrated CO_2 gases. (**b**) The $\Delta_{49\,(raw)}$ values of the carbonate-derived CO_2 plot between the $\Delta_{49\,(raw)}$ values of the presumably uncontaminated equilibrated CO_2 gases. (**c**) $\Delta_{49\,(raw)}$ vs $\Delta_{48\,(raw)}$. The shot noise limit for Δ_{48} and Δ_{49} are 0.023‰ and 0.224‰, respectively[14].

All $\Delta_{49\,(raw)}$ values of the carbonates fall within the range of the $\Delta_{49\,(raw)}$ values of the equilibrated gases, indicating no additional contamination of the investigated solids relative to the equilibrated gases. The absolute scatter of replicate data is clearly higher than the corresponding shot noise limit. However, for each sample, there is a lack of correlation between $\Delta_{48\,(raw)}$ and $\Delta_{49\,(raw)}$ values, demonstrating that, whatever evokes the scatter in $\Delta_{49\,(raw)}$ values, has not affected $\Delta_{48\,(raw)}$ values.

Supplementary References

1 Coplen, T. B. Calibration of the calcite–water oxygen-isotope geothermometer at Devils Hole, Nevada, a natural laboratory. *Geochim. Cosmochim. Acta* **71**, 3948-3957 (2007). https://doi.org/10.1016/j.gca.2007.05.028

2 Hansen, M., Scholz, D., Schöne, B. R. & Spötl, C. Simulating speleothem growth in the laboratory: Determination of the stable isotope fractionation ($\delta^{13}C$ and $\delta^{18}O$) between H_2O, DIC and $CaCO_3$. *Chem. Geol.* **509**, 20-44 (2019). https://doi.org/10.1016/j.chemgeo.2018.12.012

3 Schmidt, G. A., Bigg, G. R. & Rohling, E. J. *Global Seawater Oxygen-18 Database - v1.21*. (1999).

4 Storz, D., Gischler, E., Fiebig, J., Eisenhauer, A. & Garbe-Schonberg, D. Evaluation of oxygen isotope and Sr/Ca ratios from a Maldivian scleractinian coral for reconstruction of climate variability in the northwestern Indian Ocean. *Palaios* **28**, 42-55 (2013). https://doi.org/10.2110/palo.2012.p12-034r

5 Kim, S.-T., Mucci, A. & Taylor, B. E. Phosphoric acid fractionation factors for calcite and aragonite between 25 and 75 °C: Revisited. *Chem. Geol.* **246**, 135-146 (2007). https://doi.org/10.1016/j.chemgeo.2007.08.005

6 Spötl, C. & Cheng, H. Holocene climate change, permafrost and cryogenic carbonate formation: insights from a recently deglaciated, high-elevation cave in the Austrian Alps. *Clim. Past* **10**, 1349-1362 (2014). https://doi.org/10.5194/cp-10-1349-2014

7 Žák, K. *et al.* in *Ice Caves* (eds Aurel Perşoiu & Stein-Erik Lauritzen) Ch. 6, 123-162 (Elsevier, 2018).

8 Kluge, T. *et al.* Clumped isotope thermometry of cryogenic cave carbonates. *Geochim. Cosmochim. Acta* **126**, 541-554 (2014). https://doi.org/10.1016/j.gca.2013.11.011

9 Petersen, S. V. *et al.* Effects of improved ^{17}O correction on interlaboratory agreement in clumped isotope calibrations, estimates of mineral-specific offsets, and temperature dependence of acid digestion fractionation. *Geochem. Geophys. Geosyst.* **20**, 3495-3519 (2019). https://doi.org/10.1029/2018GC008127

10 Fernandez, A. *et al.* A reassessment of the precision of carbonate clumped isotope measurements: Implications for calibrations and paleoclimate reconstructions. *Geochem. Geophys. Geosyst.* **18**, 4375-4386 (2017). https://doi.org/10.1002/2017gc007106

11 Fiebig, J. *et al.* Combined high-precision Δ_{48} and Δ_{47} analysis of carbonates. *Chem. Geol.* **522**, 186-191 (2019). https://doi.org/10.1016/j.chemgeo.2019.05.019

12 Hill, P. S., Tripati, A. K. & Schauble, E. A. Theoretical constraints on the effects of pH, salinity, and temperature on clumped isotope signatures of dissolved inorganic carbon species and precipitating carbonate minerals. *Geochim. Cosmochim. Acta* **125**, 610-652 (2014). https://doi.org/10.1016/j.gca.2013.06.018

13 Bernasconi, S. M. *et al.* Reducing uncertainties in carbonate clumped isotope analysis through consistent carbonate-based standardization. *Geochem. Geophys. Geosyst.* **19**, 2895-2914 (2018). https://doi.org/10.1029/2017gc007385

14 Merrit, D. A. & Hayes, J. M. Factors controlling precision and accuracy in isotope-ratio-monrtoring mass spectrometry. *Anal. Chem.* **66**, 2336-2347 (1994). https://doi.org/10.1021/ac00086a020
