# Supplementary material for: Dual clumped isotope thermometry resolves kinetic biases in carbonate formation temperatures
Source: Nat Commun. 2020 Aug 10;11:4005. doi: 10.1038/s41467-020-17501-0 (PMC7418028; doi:10.1038/s41467-020-17501-0)
Supplement: Supplementary file 2 — Description of Additional Supplementary Files [file 41467_2020_17501_MOESM2_ESM.pdf]

Description of Additional Supplementary Files

File Name: Supplementary Data 1

Description: The file contains data for all replicate analyses of equilibrated gases, and carbonate reference material (ETH 1, ETH 2, and ETH 3) for the April–August 2019 measurement period. The raw isotope values in this file are calculated with a uniform m/z 47.5 scaling factor of -1 for m/z 47–49. The values within were used to calculate the long-term Δ47 (CDES90) and Δ48 (CDES90) values for the standards.

File Name: Supplementary Data 2

Description: The file contains data for all replicate analyses of equilibrated gases, carbonate reference material, and samples for the April–August 2019 measurement period. Replicates in this file are identical to those in Supplementary Data 1, except that here, the non-linearity corrected raw isotope values are calculated with empirically determined m/z 47.5 scaling factors.

File Name: Supplementary Data 3.

Description: The file contains data for all replicate analyses of equilibrated gases, carbonate reference material, and samples for the September– December 2019 measurement period. Non-linearity corrected raw isotope values are calculated using empirically determined m/z 47.5 scaling factors. 2020-06-21 Seite 2 von 2

File Name: Supplementary Data 4

Description: The file contains data for all replicate analyses of equilibrated gases, carbonate reference material, and samples for the January– March 2020 measurement period. Non-linearity corrected raw isotope values are calculated using empirically determined m/z 47.5 scaling factors.

File Name: Supplementary Data 5

Description: The file contains the model output data simulating disequilibrium effects in δ18O, Δ63, and Δ64 for the cold-water coral, the warm-water coral, and the synthetic speleothem. The model parameters are detailed in the Methods section of the manuscript.
